# Molecular Assembly Unlocks Dual‐Defect Synergy in Carbon Nitride for Efficient H_2_O_2_ Photosynthesis

**DOI:** 10.1002/advs.202517957

**Published:** 2025-11-08

**Authors:** Xiaolin Sun, Pengfei Tian, Jinye Li, Minghui Zhu, Jing Xu, Fu‐Zhen Xuan

**Affiliations:** ^1^ State Key Laboratory of Green Chemical Engineering and Industrial Catalysis School of Chemical Engineering East China University of Science and Technology Shanghai 200237 P. R. China; ^2^ Key Laboratory of Pressure Systems and Safety (Ministry of Education) School of Mechanical and Power Engineering East China University of Science and Technology Shanghai 200237 P. R. China; ^3^ University Engineering Research Center of Green Chemical New Materials School of Chemistry and Chemical Engineering Guangxi University Nanning Guangxi 530004 P. R. China

**Keywords:** defected carbon nitride, hydrogen peroxide synthesis, molecular assembly‐molten salt coupling, Photocatalytic oxygen reduction

## Abstract

While promising for photocatalytic hydrogen peroxide (H_2_O_2_) production, the performance of graphitic carbon nitride (g‐C_3_N_4_) is curtailed by a central synthesis paradox: the mutually exclusive conditions required to simultaneously create its most effective dual active sites—nitrogen vacancies and cyano groups. Herein, this paradox is resolved with a molecular assembly‐molten salt coupling strategy, a precise bottom‐up approach enabling the one‐step, synergistic creation of K‐doped g‐C_3_N_4_ with both defect types. This photocatalyst achieves an exceptional H_2_O_2_ production activity of 2.65 mmol·g^−1^·h^−1^, which is 6.2 and 3.0 times higher than that of pristine and physically‐ground K‐doped g‐C_3_N_4_, respectively. Characterization and theoretical calculations reveal that molecular assembly promotes K^+^ interlayer embedding to facilitate charge migration, while the dual defects exhibit functional complementarity: nitrogen vacancies enhance O_2_ adsorption, and cyano groups facilitate proton coupling. In situ analysis also confirms an easier O_2_ activation effect and a lowered energy barrier for ^*^OOH formation, ensuring high selectivity via a two‐step, single‐electron pathway. This study not only offers a route to rationally engineer dual‐defect sites in carbon nitride but also provides a generalizable strategy for designing other advanced photocatalysts.

## Graphical abstract

A long‐standing puzzle in making catalysts for hydrogen peroxide is broken. The innovation is a synthesis strategy that creates two synergistic defect sites (cyano defects and N vacancy defects). This synergy enables highly efficient and selective H_2_O_2_ production.

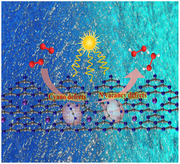

## 1 Introduction

In the context of the global energy structure transition and “carbon neutrality” strategies, the development of green and sustainable energy carriers and chemical synthesis pathways is paramount. Hydrogen peroxide (H_2_O_2_), as a high‐energy‐density liquid fuel and environmentally friendly oxidant, demonstrates immense application potential in the fields of energy, chemicals, and environmental protection.^[^ ^1^, ^2^ ^]^ However, more than 90% of global H_2_O_2_ production relies on the energy‐intensive and highly polluting anthraquinone process. Alternatively, the direct thermal synthesis of H_2_O_2_ from H_2_ and O_2_, while promising, requires precious metal catalysts (e.g., Pd, Au) and faces significant bottlenecks. These include the risk of gas mixture explosion, low product selectivity, and increased costs from inert supports, exchange membranes, and additional gases.^[^ ^3^, ^4^ ^]^ Therefore, developing efficient, safe, and green on‐site H_2_O_2_ synthesis technology is urgent. The preparation of H_2_O_2_ based on photocatalysis has attracted much attention in recent years. The process is simple and mild, requiring only water, oxygen, and sunlight. It does not produce toxic by‐products, making it safe and sustainable. Graphite‐phase carbon nitride (g‐C_3_N_4_) is a metal‐free conjugated semiconductor that has been widely employed in photocatalysis due to its low cost, non‐toxicity, and facile synthesis and modification. However, the photocatalytic activity of pristine g‐C_3_N_4_ is fundamentally hindered by a rapid charge carrier recombination and a paucity of suitable active sites. Therefore, the modification of g‐C_3_N_4_ is required to overcome these inherent limitations.

The doping of alkali metal ions (e.g., K) has been demonstrated to be a viable strategy for modification of g‐C_3_N_4_. This process is typically achieved by the co‐calcination of an intimately grinding mixture of g‐C_3_N_4_ precursors and metal salts.^[^ ^5^ ^]^ This modification can enhance photocatalytic performance by broadening the photoresponse range, optimizing the band structure, promoting interlayer charge transfer, and possibly generating cyano‐rich (─C≡N) groups with stronger reducing ability.^[^ ^6^, ^7^ ^]^ These cyano groups function as crucial active sites that facilitate proton adsorption and promote the subsequent reaction. However, the enhancement in carrier separation efficiency achieved via K‐doping alone is constrained by an insufficient intrinsic structural defects, particularly a paucity of nitrogen (N) vacancy sites capable of modulating the reaction pathway. Although the incorporation of cyano groups enhances reaction rates, this approach fails to adequately address the issue of active site singletization. To maximize photocatalytic efficiency, an ideal g‐C_3_N_4_ architecture would synergistically combine the enhanced O_2_ activation from nitrogen vacancies with the superior proton‐coupling ability of cyano groups. However, conventional synthesis routes face a critical paradox: the inert atmosphere required to create stable nitrogen vacancies inevitably leads to the thermal destruction of valuable cyano groups, making it challenging to optimize both defect sites simultaneously.^[^ ^8^, ^9^ ^]^

In this work, we introduce a generalizable bottom‐up strategy—molecular assembly‐molten salt coupling—for the one‐pot synthesis of the modified g‐C_3_N_4_ catalyst. This approach leverages molecular‐level pre‐organization within the precursor solution to guide the precise, simultaneous formation of dual defects and K^+^ doping in air. It fundamentally circumvents the limitations of non‐homogeneous solid‐state reactions and resolves the aforementioned synthesis conflict, paving a new way for advanced active‐site engineering. The process involves the one‐pot calcination in air of a melamine, cyanuric acid, and KCl assembly, which synergistically achieves the in situ K^+^ doping and the formation of dual defects (N vacancies and ─C≡N groups). Specifically, it demonstrated a high photocatalytic synthesis of H_2_O_2_ (2.65 mmol g^−1^ h^−1^) under visible light (λ ≥ 420 nm). Experimental characterization and theoretical calculations confirm that the catalyst is doped with K ions, which are used as an interlayer skeleton for transferring electrons and forming N vacancies and cyano groups simultaneously within the induced layer, both of which optimize the O_2_ adsorption activation and proton coupling pathways, respectively. This strategy is readily extendable to alkaline metal systems such as Na/Cs, providing a new paradigm for active‐site engineering of g‐C_3_N_4_, with potential applications in fields such as Fenton‐like advanced oxidation.

## 2 Results and Discussion

### 2.1 Synthesis of KMACN‐A and Photocatalytic Performance

**Scheme 1:**
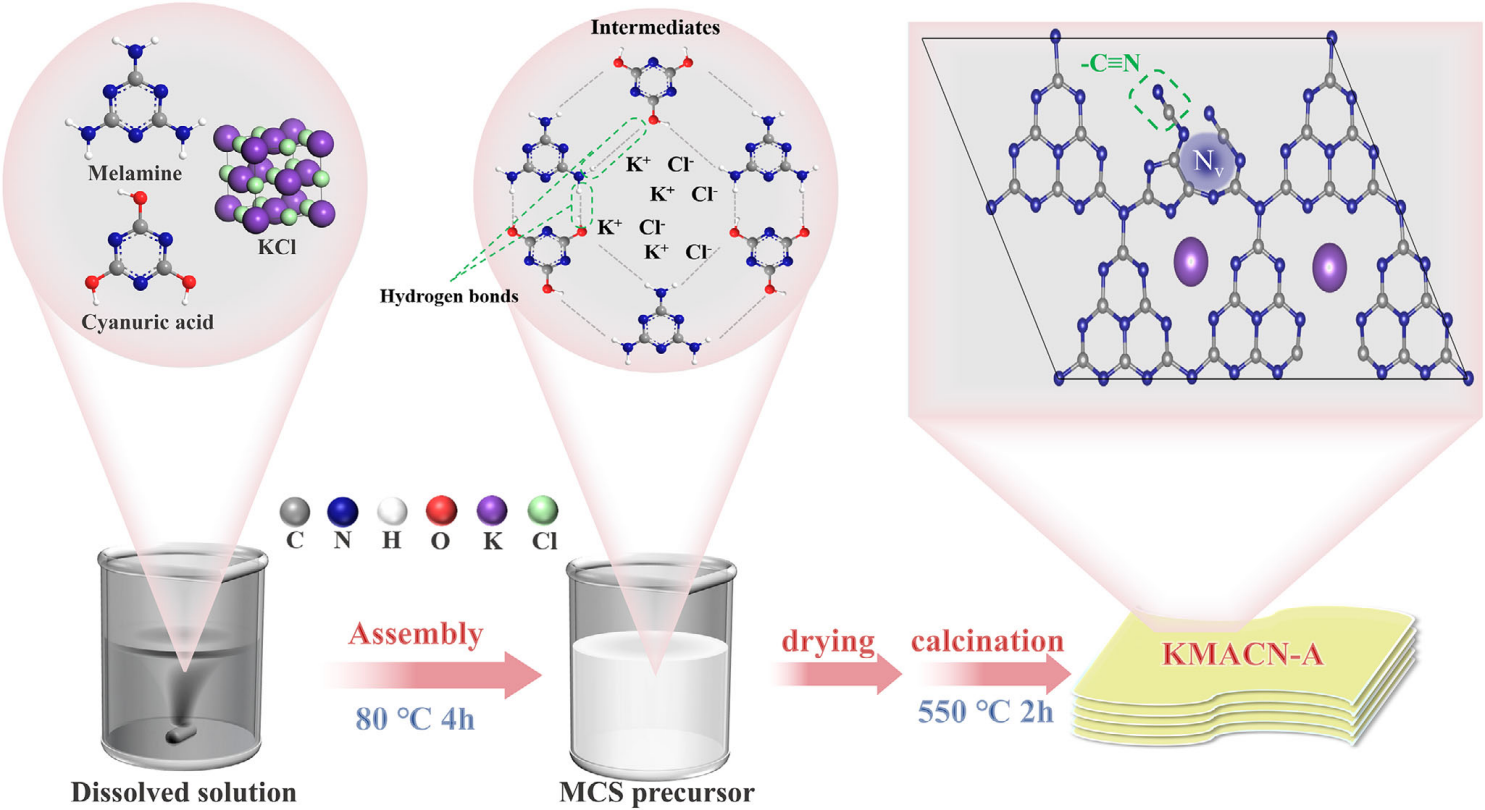
Schematic illustration of the synthesis molecular assembly‐molten salt coupling process of KMACN‐A.

As depicted in **Scheme** **1**, a molecular assembly‐molten salt coupling strategy was employed to in situ dope of potassium into the supramolecular network of the carbon nitride precursor. During the stirring process, melamine and cyanuric acid were self‐assembled via hydrogen‐bonding. Meanwhile, K^+^ and Cl^−^ ions, derived from KCl dispersed throughout the assembly, facilitating the formation of the supramolecular compound. The loosely connected supramolecular precursors served as a 3D framework during subsequent calcination. After thermal polymerization in air, KMACN‐A was obtained. For comparison, K‐doped samples (KMCN‐G/KMACN‐G) were also prepared using the conventional physical grinding method.

**Figure 1:**
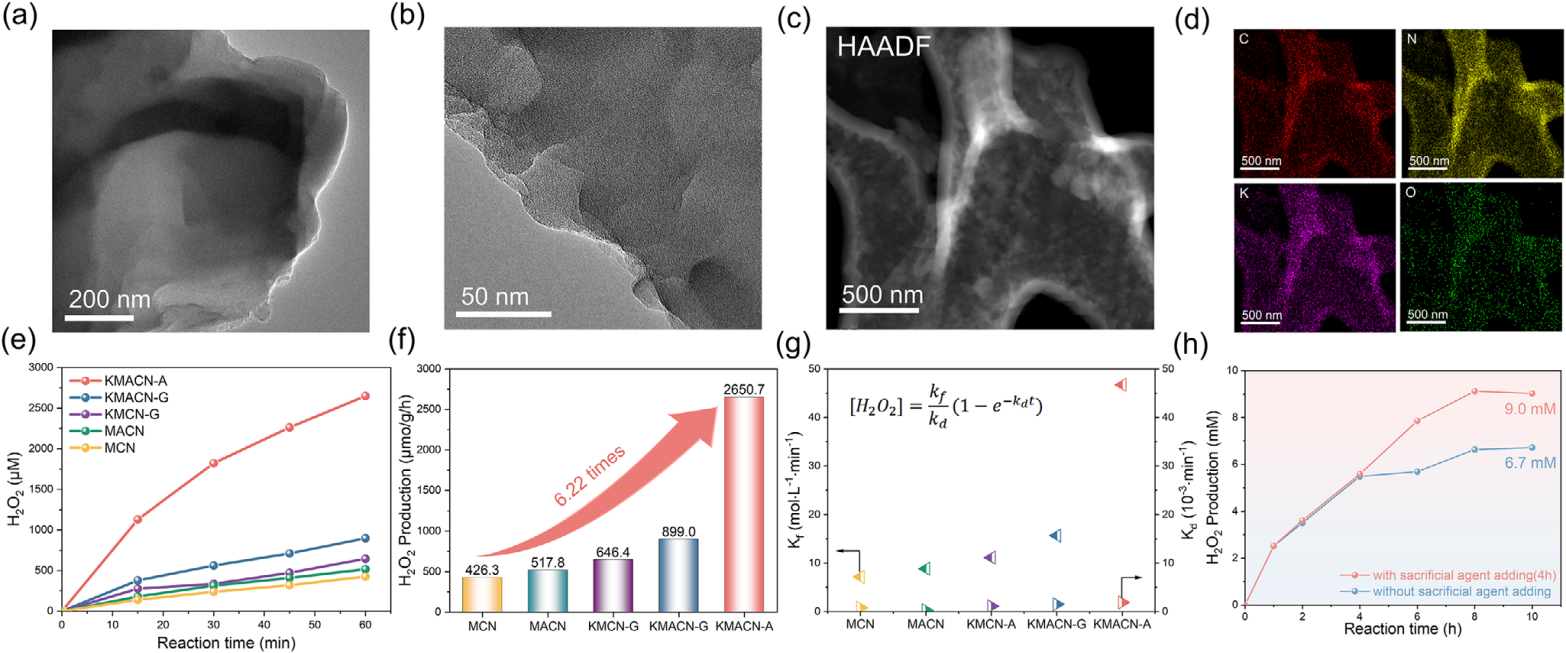
a,b) TEM micrograph, c) HRTEM images of KMACN‐A. d) EDS elemental mapping of KMACN‐A. e) Photocatalytic H_2_O_2_ production, f) Corresponding rate over as‐prepared catalysts (λ ≥ 420 nm), g) Rates of H_2_O_2_ formation (k_f_, left) and H_2_O_2_ decomposition (k_d_, right) for various CN catalysts. h) Time course of H_2_O_2_ evolution over 10 h.

Scanning electron microscopy (SEM) revealed distinct morphological differences among the polymeric carbon nitride samples prepared by various methods.^[^ ^10^ ^]^ As shown in Figure S2 (Supporting Information), MCN exhibits a typical fluffy, layered structure. In contrast, the K‐doped materials display block‐like and densely packed layered structures. The material structures were further investigated by transmission electron microscopy (TEM).^[^ ^11^ ^]^ As seen in **Figure** **1**a–d, KMACN‐A consists of nanosheets with irregular edges and laterally curled 2D structures. Elemental mapping and spherical aberration transmission electron microscopy of both KMACN‐G (Figures S3 and S4, Supporting Information) and KMACN‐A reveal a uniform distribution of C, N, O, and K. These results confirm the successful incorporation of K into the materials by both preparation methods.

To investigate the effect of different preparation methods on K‐doping, the photocatalytic H_2_O_2_ production performance of the samples was evaluated. As depicted in Figure 1e,f, the KMACN‐A exhibited significantly enhanced H_2_O_2_ production performance. It achieved an optimal H_2_O_2_ generation rate of 2.65 mmol g^−1^ h^−1^, a value 6.22 and 2.95 times that of MCN (0.43 mmol g^−1^ h^−1^) and KMACN‐G (0.89 mmol g^−1^ h^−1^), respectively. The photocatalytic performance of KMACN‐A for H_2_O_2_ synthesis under visible light was higher than that of most reported photocatalysts in Table S1 (Supporting Information). The respective generation and degradation rates of H_2_O_2_ for the different catalysts are shown in Figure 1g. The net accumulation of H_2_O_2_ is determined by the kinetic competition between its formation (rate constant k_f_) and decomposition (rate constant k_d_). Accordingly, the net H_2_O_2_ concentration can be described by Equation (1).

(1) $$\left[H_{2}O_{2}\right]=\left(\frac{k_{f}}{k_{d}}\right)\;\left\{1-\exp\left(-k_{d}\times t\right)\right\}$$

The reaction kinetics can be obtained by assuming that the corresponding k_f_ is a zero‐order reaction and k_d_ is a first‐order reaction. The fitting results indicate that KMACN‐A possesses a high formation rate constant (k_f_) and a relatively low decomposition rate constant (k_d_). This kinetic profile is consistent with its superior overall H_2_O_2_ production rate compared to the other catalysts.

Moreover, the long‐term photocatalytic stability of KMACN‐A for H_2_O_2_ production was evaluated (Figure 1h). In an initial 10 h test with a single dose of sacrificial agent, the catalyst produced 6.7 mm of H_2_O_2_. The observed decrease in the reaction rate was attributed to the depletion of the sacrificial agent, which limited the proton supply for H_2_O_2_ synthesis. To verify this, a second experiment was conducted where an additional amount of sacrificial agent was injected after 4 h. This approach yielded a higher concentration of 9.0 mm H_2_O_2_ over a 10 h period. The H_2_O_2_ concentration was observed to decrease after reaching a peak at the 8 h mark. This decline is attributed to the decomposition of H_2_O_2_, which becomes more significant as the product accumulates in the solution (Figure S5, Supporting Information). These results demonstrate the robust stability of the catalyst and suggest its significant potential for the on‐site photocatalytic synthesis of H_2_O_2_ for applications such as the Fenton‐like advanced oxidation process.

### 2.2 Physical Structure

**Figure 2:**
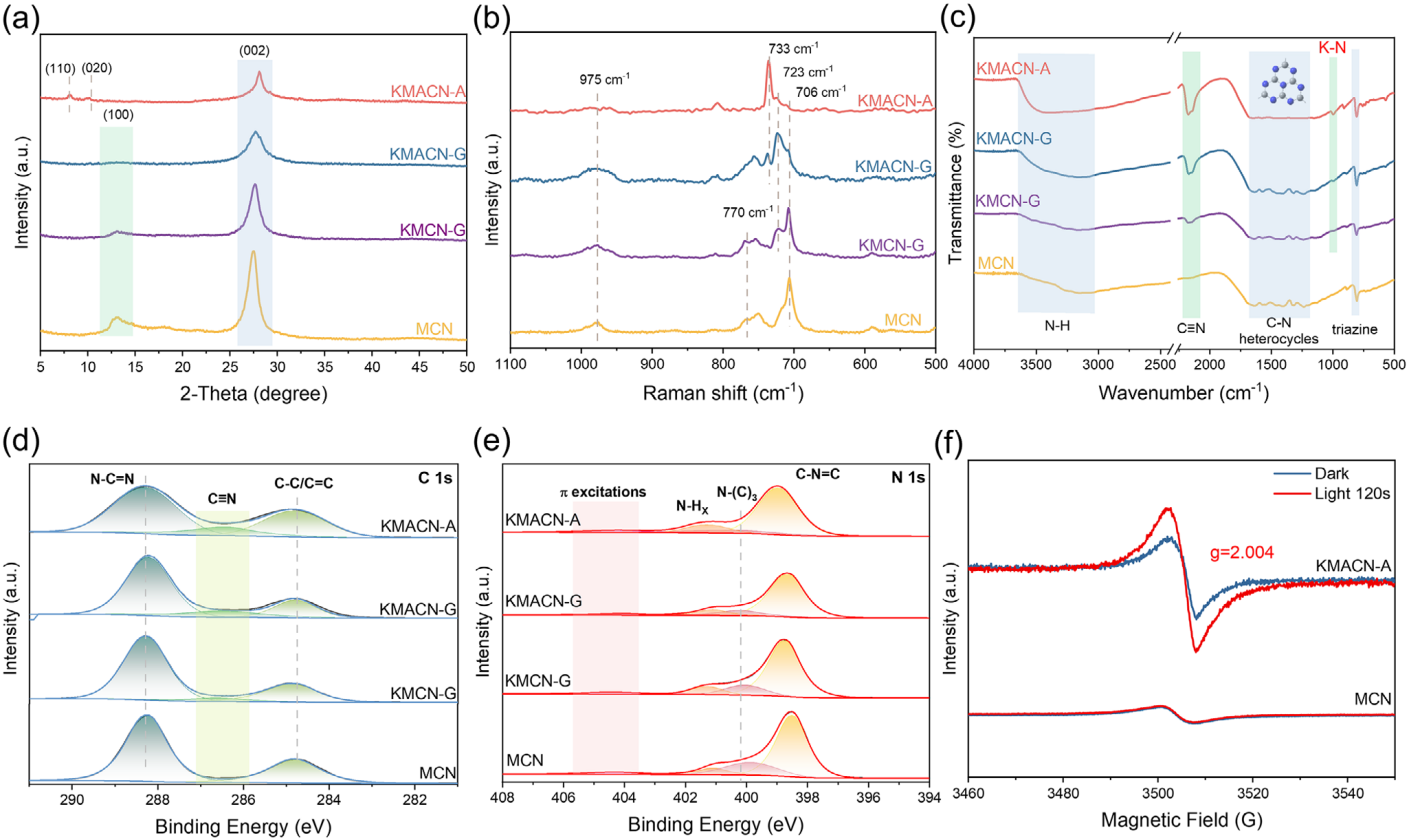
a) The XRD pattern, b) Raman spectra, c) FT‐IR spectra, d) High‐resolution C 1s XPS, e) High‐resolution N 1s XPS of MCN and various K‐CN. f) ESR spectra of MCN and KMACN‐A under dark and light conditions.

To characterize the crystal structure of the prepared catalysts, X‐ray diffraction (XRD) and Raman spectroscopy were performed. The XRD patterns of all samples (**Figure** **2**a) exhibit similar diffraction peaks, suggesting that the fundamental crystal structure of carbon nitride was preserved regardless of the preparation method. The two typical diffraction peaks of pristine MCN at 13.1°and 27.6° can be attributed to the in‐plane stacked (100) facet and interlayer stacked (002) facet of g‐C_3_N_4_ sheets.^[^ ^12^, ^13^ ^]^ Compared to pristine MCN, the addition of cyanuric acid (C_3_N_3_(OH)_3_) exhibits weaker peak intensities and broader full‐width at half maximum (FWHM) values, as detailed in Table S2 (Supporting Information). This phenomenon is attributed to the different reactivities of the hydroxyl groups (‐OH) of cyanuric acid and the amino groups (‐NH_2_) of melamine during thermal polymerization. This difference in reactivity promotes a disordered polycondensation pathway, thereby reducing the crystallinity of the catalyst.

The incorporation of potassium salts induces further structural modifications in the catalyst. For the KMACN‐G and KMACN‐A samples, the diffraction peak corresponding to the (100) plane is diminished or absent, indicating a disruption of the in‐plane stacking of the heptazine units. Notably, for KMACN‐A, new diffraction peaks emerge at 8.0° and 10.1°, which correspond to the (110) and (020) planes, respectively.^[^ ^14^ ^]^ These changes suggest that K^+^ induces a remodeling of the heptazine ring arrangement, leading to the formation of an in‐plane folded framework. Concurrently, the intercalation of K^+^ causes the main (002) diffraction peak to shift from 27.6° to 28.1°.^[^ ^15^ ^]^ According to Bragg's law, this corresponds to a decrease in the interlayer spacing from 0.326 to 0.320 nm. This finding is corroborated by theoretical calculations (Figure S6, Supporting Information), which show that the intercalation of K^+^ ions (diameter: 0.276 nm), being smaller than the interlayer spacing of g‐C_3_N_4_ (0.326 nm), effectively narrows this gap. As a probe highly sensitive to local symmetry, Raman spectroscopy further corroborates this structural distortion in Figure 2b. The peaks near 706, 770, and 975 cm^−1^ are attributed to the in‐plane bending vibration of the tri‐s‐triazine ring, the heptazine ring stretching vibration, and the symmetric N breathing mode of the heptazine unit in carbon nitride, respectively.^[^ ^16^, ^17^, ^18^ ^]^ For KMACN‐G, the characteristic peak (706 cm^−1^) exhibits significant perturbation and shifting, accompanied by the emergence of new vibrational modes (733 cm^−1^). These changes in peak intensity are even more pronounced in KMACN‐A. These changes reflect a severe disruption of local symmetry, resulting from the formation of K─N bonds and defect structures.

To investigate the structural origins of the differences in catalytic activity, the defect structures of the catalysts were identified. As shown in Figures 2c and S7 (Supporting Information), The FT‐IR characteristic peaks located in the region of 810 and 1200–1600 cm^−1^ are attributed to the bending and stretching vibration modes characteristic of aromatic CN heterocycles,^[^ ^5^, ^19^ ^]^ while the peaks in the 2900–3000 cm^−1^ region represent the N‐H vibrations in the surface‐terminal group (‐NH_2_).^[^ ^20^ ^]^ The peak at 998 cm^−1^ is assigned to the symmetric and asymmetric vibrations of the NC_2_ bond in the metal‐NC_2_ moiety, indicating the formation of K─N bonds after K‐doping.^[^ ^21^ ^]^ Interestingly, a new peak emerged at 2172 cm^−1^, which is attributed to the formation of cyano (─C≡N) groups. During the high‐temperature synthesis, KCl may react with hydrogen (e.g., ‐NH‐ groups) in g‐C_3_N_4_ to generate HCl, which locally etches the framework, creates unsaturated sites, and introduces cyano groups into the carbon nitride structure.^[^ ^6^, ^14^, ^20^ ^]^ And the catalyst was characterized after the reaction to confirm its structural stability, as its original structure is largely retained (Figures S8–S13, Supporting Information). Moreover, the high‐resolution C 1s XPS spectra (Figure 2d; Figure S14, Supporting Information) display characteristic binding energies at 284.8 and 288.3 eV, which are assigned to C─C/C═C bonds in graphitic carbon and aromatic N─C═N structures in sp^2^‐hybridized carbon, respectively. The emergence of a new peak at 286.6 eV further confirms the formation of cyano (─C≡N) groups after K‐doping.

**Figure 3:**
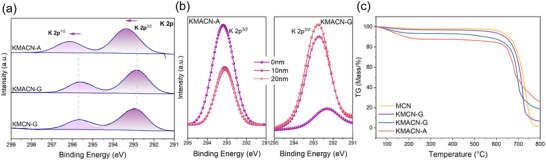
a) High‐resolution K 2p XPS of MCN and various K‐CN. b) High‐resolution K 2p XPS spectra of KMACN‐G/A at different depths. c) TG curves of MCN and various K‐CN.

In addition, the N 1s spectra (**Figure** **3**e) consistently exhibit three peaks at 398.7, 400.2, and 401.3 eV, corresponding to two‐coordinated C─N═C, three‐coordinated N‐(C)_3_, and terminal N‐H_X_, respectively.^[^ ^7^, ^14^, ^22^ ^]^ Notably, the N/C ratio in KMACN‐A (0.96) is markedly lower than that in MCN (1.04), conversely, the N_2C_/N_3C_ ratio (5.50) is significantly higher than in MCN (3.14), as shown in Table S3 (Supporting Information). These results confirm the formation of nitrogen vacancies arising from the loss of N atoms from N‐(C)_3_ sites.^[^ ^8^, ^23^, ^24^, ^25^ ^]^ The formation of nitrogen vacancies was further substantiated by electron paramagnetic resonance (EPR) spectroscopy (Figure 2g; Figure S16, Supporting Information). The strong Lorentzian signal observed at g = 2.004 for KMACN‐A is a characteristic fingerprint of unpaired electrons localized at nitrogen vacancies.^[^ ^26^ ^]^

The K 2p spectra (Figure 3a) exhibit two peaks centered at 293.9 and 295.7 eV, confirming the presence of potassium.^[^ ^27^ ^]^ Additionally, a notable shift of the peak position towards higher binding energies is observed in KMACN‐A. The reduced electron density of K ions may be due to the aggregation of more K at the surface, thereby forming more K─N bonds (Table S4, Supporting Information). This strong interaction results in a greater electron withdrawal from the K^+^ ions by the more electronegative nitrogen atoms, thus reducing the electron cloud density around K and causing a shift to a higher binding energy. To elucidate the origin of these differences, the spatial distribution of potassium elements was investigated in depth using the XPS Ar etching with a depth increment of 10 nm (Figure 3b). The analysis revealed distinct elemental distribution patterns resulting from the two preparation methods. With increasing etching depth, the KMACN‐A sample prepared by supramolecular assembly shows significant potassium surface enrichment features, whereas the KMACN‐G sample prepared by the physical mixing method mainly exhibits bulk phase doping.

The fundamental reason for this discrepancy in macroscopic distribution may stem from the substantial difference in synthetic pathways at the atomic and molecular levels. The molecular assembly strategy relies on the pre‐construction of an ordered supramolecular network in the precursor solution, driven by hydrogen‐bonding. This process likely facilitates the uniform dispersion and in situ integration of potassium ions within the precursor network prior to polymerization. This molecular‐scale pre‐organization strategy potentially provides a precise guidance for the subsequent high‐temperature polymerization reaction. Consequently, during the assembly process, K^+^ ions are likely encapsulated within the gaps of the hydrogen‐bonded network. During the subsequent calcination, K^+^ ions near the surface can form coordination bonds with functional groups (e.g., –NH_2_), thereby immobilizing them. In contrast, the unanchored K^+^ ions from the interior may escape due to thermal perturbation. Ultimately, this pre‐positioning allows K^+^ ions to embed effectively within the g‐C_3_N_4_ layers over very short diffusion distances, which in turn can accelerate the formation of structural defects.

In contrast, the physical mixing method involves non‐homogeneous solid‐phase reactions, where interactions are likely constrained to a limited number of particle interfaces. During high‐temperature treatment, because the melting point of the K salt typically exceeds the polymerization temperature of carbon nitride, the g‐C_3_N_4_ network is presumed to form first. This reaction sequence means that K^+^ ions can only be incorporated into the bulk‐phase lattice or between particles via stochastic, long‐range diffusion. Consequently, this mechanism significantly restricts the ability of K^+^ ions to modify the interlayer structure. The proposed mechanism is supported by thermogravimetric analysis (TG) results (Figure 3c; Figure S17, Supporting Information). For the KMACN‐A sample, the increased proportion of bound water observed below 200 °C is attributed to the hydrophilicity of the surface‐enriched K^+^ layer.^[^ ^28^ ^]^ Furthermore, KMACN‐A exhibits a lower onset thermal decomposition temperature (500–700 °C) for the CN backbone. This reduced thermal stability is attributed to a combination of factors: the presence of surface‐enriched K^+^ ions and the disruption of skeletal integrity by numerous lattice defects.^[^ ^29^, ^30^ ^]^

### 2.3 Energy Band Structure and Charge Transfer Properties

**Figure 4:**
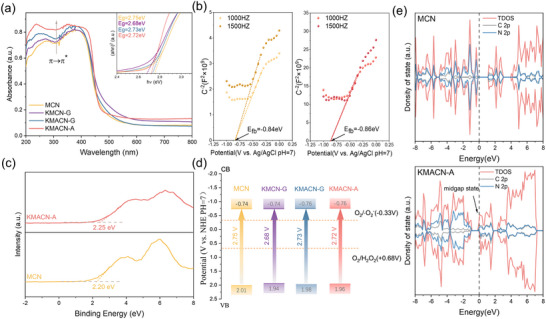
a) UV–vis DRS spectra of MCN and various K‐CN (inset: Tauc plot of MCN and various K‐CN). b) Mott–Schottky plots of MCN and KMACN‐A in 0.5 m Na_2_SO_4_. c) Valence band‐XPS of MCN and KMACN‐A from XPS spectra. d) Schematic illustration of the energy level structure of MCN and various K‐CN. e) Calculated density of states (DOS) of MCN and KMACN‐A.

To gain deeper insight into the optical properties of K‐doped g‐C_3_N_4_ catalysts prepared by different methods, a series of characterizations and calculations was conducted to deduce the detailed band structures of the photocatalysts. The UV–vis diffuse reflectance spectra (DRS, **Figure** **4**a) show that K‐doping clearly enhances the light‐harvesting ability compared to MCN. This is evidenced by a significant increase in absorbance above 550 nm and a marked redshift of the absorption edge. This phenomenon can mainly be attributed to π → π^*^ electronic transitions within the conjugated aromatic rings, as well as a large number of n → π^*^ transitions of lone‐pair electrons on the cyano groups.^[^ ^31^ ^]^ Based on the above UV–vis DRS results, the corresponding energy bandgaps (Eg) were further calculated using the transformed Kubelka–Munk function, and the Tauc plots indicate that the Eg values for MCN, KMCN‐G, KMACN‐G, and KMACN‐A are 2.75, 2.68, 2.73, and 2.72 V, respectively. Despite KMCN‐G exhibiting high visible‐light absorption and a relatively narrow bandgap, its lower density of internal defects is not conducive to efficient charge separation. Consequently, even though it absorbs more photons, the excessively narrow bandgap enhances the Coulombic attraction between photogenerated electrons and holes, which ultimately reduces the efficiency of charge carrier separation and providing insufficient driving force for the redox reactions.

Moreover, as illustrated in the Mott–Schottky plots (Figure 4b; Figure S18, Supporting Information), the positive slope confirms that all samples are n‐type semiconductors, and their flat band potentials (E_fB_) were calculated using E_fB_ (vs NHE) = E_fb_ (Ag/AgCl) + 0.197 eV, resulting in values of ‐0.84, ‐0.84, ‐0.85, and ‐0.86 eV for MCN, KMCN‐G, KMACN‐G, and KMACN‐A, respectively. For n‐type semiconductors, the conduction band minimum is usually ≈0.1–0.3 eV above the flat band potential.^[^ ^32^ ^]^ Thus, the conduction band positions of these samples are calculated to be ‐0.74, ‐0.74, ‐0.75, and ‐0.76 eV (vs NHE), respectively. In addition, the valence band positions were further verified by valence band X‐ray photoelectron spectroscopy (VB‐XPS, Figure 4c; Figure S19, Supporting Information). The E_VB_ of prepared materials can be calculated using the formulas of E_VB_ (vs NHE) = Φ + VB_XPS_ − 4.44 eV, where Φ and VB_XPS_ denote the work function of the XPS analyzer (4.2 eV), and the VB potential value is tested by VB‐XPS. The VB values calculated here generally align with those obtained from Mott–Schottky and UV–vis (DRS) analysis.

The energy band structure diagrams are shown in Figure 4d. It can be posited that the energy band structures of all catalysts are situated between the standard reduction potentials of O_2_^−^/·O_2_^−^ (‐0.33 V vs NHE) and O_2_/H_2_O_2_ (+0.68 V vs NHE). As demonstrated in the above research, all samples exhibited thermodynamic capacity for a two‐step one‐electron ORR reaction, suggesting that variations in their kinetics might influence performance differences.^[^ ^33^, ^34^ ^]^ In summary, these results demonstrate that the narrower bandgap induced by ionic doping not only enhances light absorption, particularly in the visible and near‐infrared wavelength regions, but also further promotes the generation of photogenerated charge carriers.

To verify the proposed energy band structure, density‐functional theory (DFT) calculations were employed to compare the electronic structure of g‐C_3_N_4_ with and without defects. Three N sites can be removed when N_3C_ vacancies are inserted. Among them, the most stable structure is identified in Figure S20d (Supporting Information) by energy calculations. The calculations show that in MCN and KMACN‐A, the top of the valence band (VBT) is primarily contributed by N 2p orbital hybridization, and the bottom of the conduction band (CBB) is mainly composed of C 2p and N 2p orbitals, which is consistent with previous studies (Figure 4e; Figure S21, Supporting Information).^[^ ^35^, ^36^ ^]^ Furthermore, an additional midgap state forms around the Fermi level of KMACN‐A, compared to MCN. This state is populated by N 2p and C 2p atoms surrounding the N vacancies and corresponds to the absorption tail extending to 800 nm, as observed in the UV–vis/DRS of KMACN‐A.^[^ ^37^ ^]^

**Figure 5:**
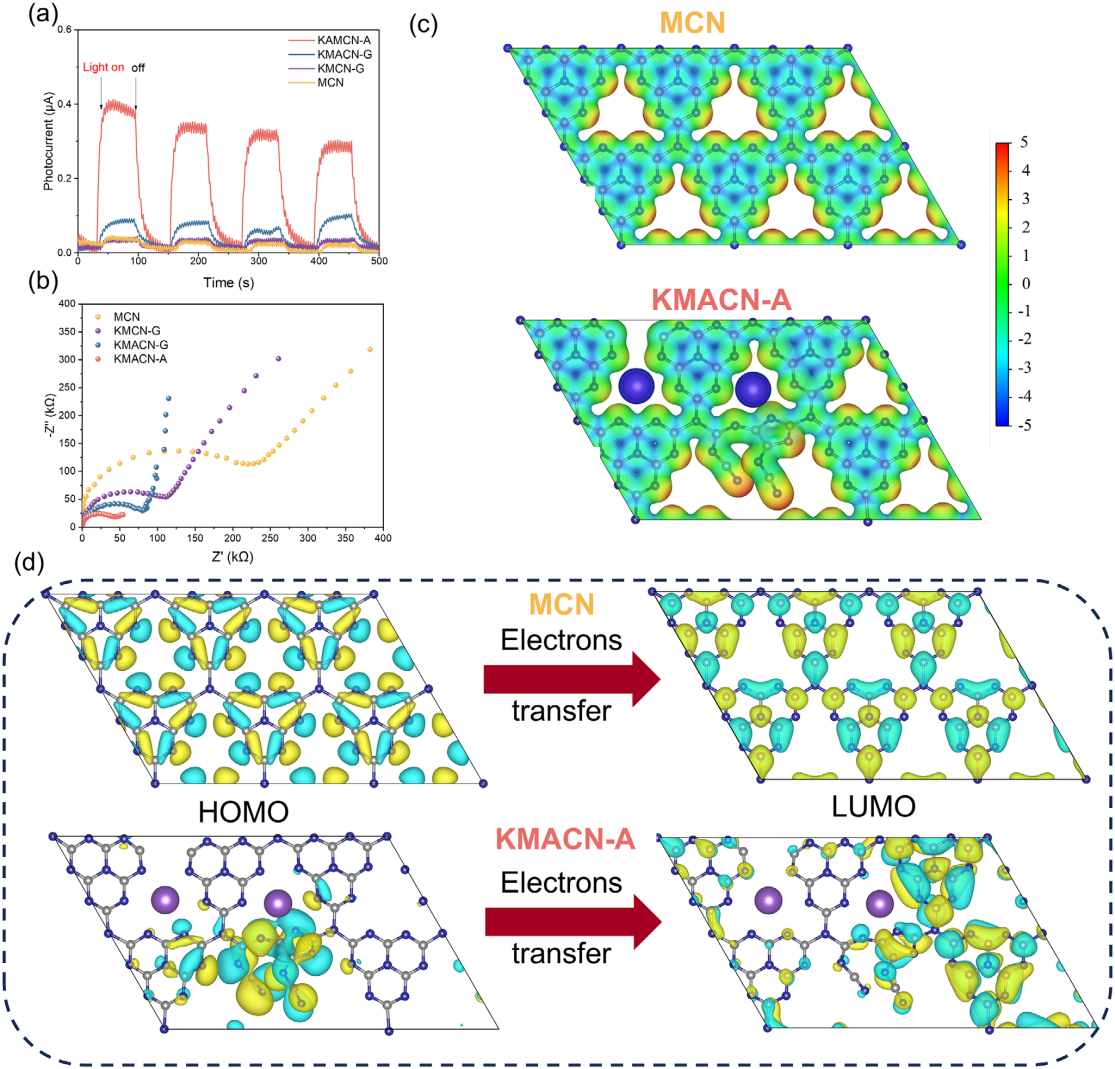
a) *I–t* curves, b) EIS Nyquist plots of the MCN and various K‐CN. c) The electrostatic potential surface distribution. d) Highest occupied molecular orbital (HOMO) and lowest unoccupied molecular orbital (LUMO) distribution of MCN and KMACN‐A.

Electrochemical measurements are regarded as a reliable technique for assessing charge dynamics.^[^ ^38^, ^39^ ^]^ **Figure** **5**a,b examined the transient photocurrent response, EIS Nyquist plots to analyze the ability of photoexcited carriers to migrate through the catalyst in a photocatalytic reaction. Notably, KMACN‐A exhibits the highest photocurrent intensity and the least extensive Nyquist radius. These observations are quantitatively substantiated by the fitted charge transfer resistance (Rct) values (Figure S22 and Table S5, Supporting Information). These results indicate that the molecular assembly method leads to superior charge carrier migration and reduced mass transfer impedance when compared to the catalysts produced by the physical grinding method with K‐doping. This phenomenon can be attributed to the introduction of cyano and N defects, which generate an internal electric field (IEF) and enhance electron transfer.^[^ ^40^, ^41^ ^]^ The presence of reactive groups effectively promotes the separation of photogenerated electrons and holes and facilitates photomobility. Meanwhile, the asymmetric electrostatic potential (Figure 5c) of KMACN‐A reflects the uneven distribution of charge density compared to MCN, which induces the dipole of molecules, promoting effective exciton dissociation.

DFT calculations were also used to investigate the effect of active sites on the microscopic charge transfer trend within the material, elucidating the microscopic mechanism behind the enhanced photocatalytic H_2_O_2_ production efficiency. As indicated in Figure 5d, the spatial distributions of the highest occupied molecular orbital (HOMO) and the lowest unoccupied molecular orbital (LUMO) for MCN reveal that, due to its homogeneous planar structure, charge carriers are uniformly delocalized across both orbitals. Conversely, K, as an alkali metal, exhibits a propensity to lose electrons, resulting in the formation of K^+^ and the development of the K─N bond. These electrons may be transferred to the π‐conjugated system of carbon nitride, thereby altering its electron cloud density and distribution. The presence of ─C≡N and N defects in the structure also results in a redistribution of charge density on KMACN‐A, leading to separated HOMO and LUMO orbitals.^[^ ^31^ ^]^ Some electron‐rich regions appear at the active site, indicating the accumulation of localized charges, which implies an efficient separation of electron–hole pairs.

### 2.4 Mechanism and Structure‐Activity Relationship of H2O2 Photosynthesis

**Figure 6:**
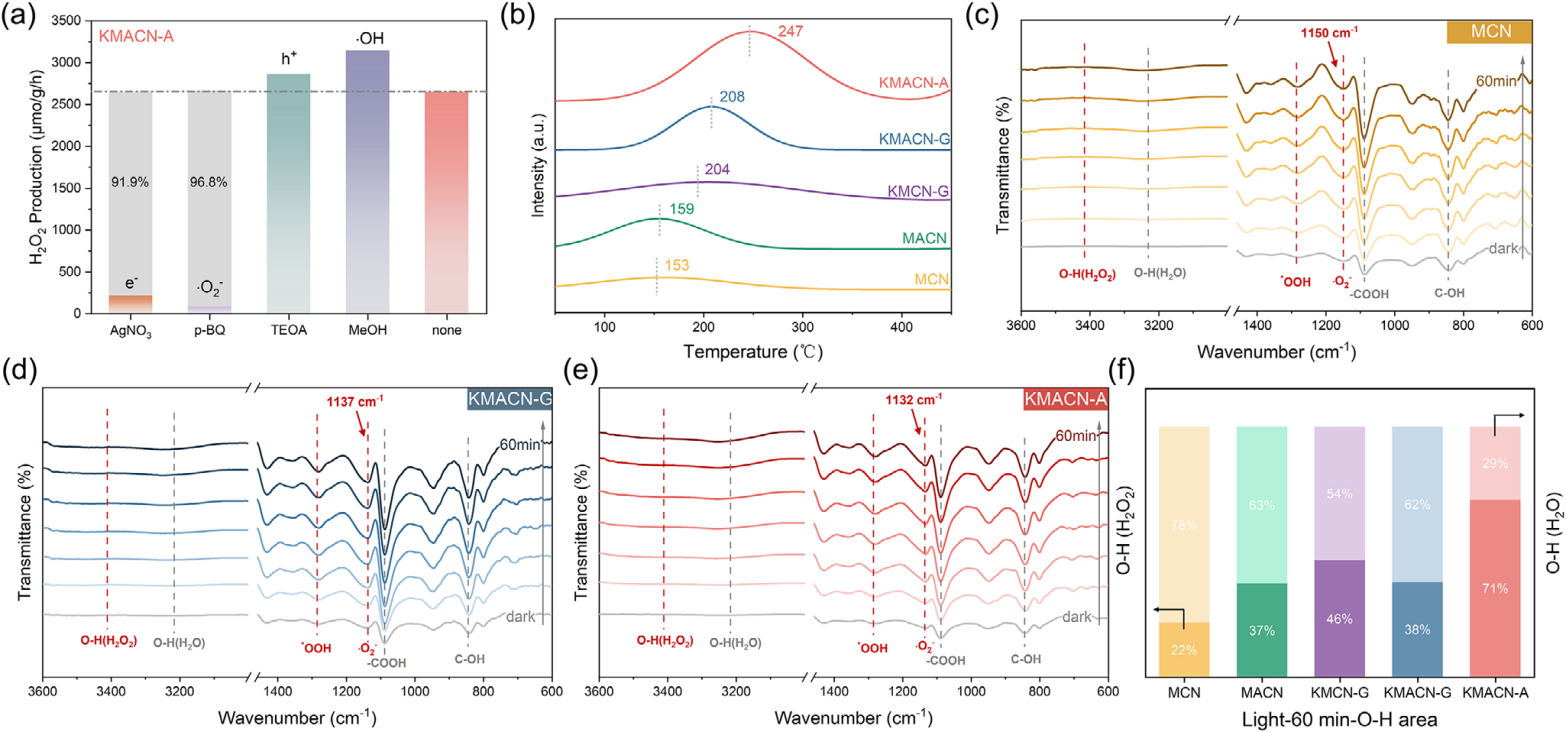
a) The photocatalytic H_2_O_2_ generation rates of KMACN‐A under different reaction gases or different sacrificial agents. b) Temperature programmed O_2_ desorption (TPD‐O_2_) profiles of all samples. In situ ATR SEIRAS spectra of photocatalysis of H_2_O_2_ from O_2_ by c) MCN, d) KMACN‐G, and e) KMACN‐A. f) Integration of the corresponding O─H bond attributed peak areas of 3000–4000 cm^−1^ from in situ ATR SEIRAS spectra of all samples at 60 min.

The primary pathway of photocatalytic H_2_O_2_ production was investigated by Free radical scavenging experiment. H_2_O_2_ production is significantly inhibited when AgNO_3_ (e^−^ scavenger) and p‐benzoquinone (·O_2_^−^ scavenger) are added in **Figure** **6**a, respectively, suggesting that photogenerated electrons and ·O_2_^−^ are the main active species in the photocatalytic H_2_O_2_ production process. This involves one electron (O_2_ + e^−^→·O_2_^−^) to form ·O_2_^−^, followed by another electron transfer to produce H_2_O_2_ (·O_2_^−^ + 2H^+^ + e^−^→H_2_O_2_).^[^ ^42^ ^]^ Notably, the addition of triethanolamine (TEOA), a hole (h^+^) scavenger, enhanced the H_2_O_2_ yield. This enhancement is attributed to the suppression of H_2_O_2_ overoxidation (H_2_O_2_ + 2h^+^ → O_2_ + 2H^+^). Similarly, the addition of methanol, a hydroxyl radical (·OH) scavenger, also increased the yield by preventing the competing four‐electron (4e^−^) oxygen reduction pathway to water (O_2_ + 4H^+^ + 4e^−^→2H_2_O), thereby favoring the desired H_2_O_2_ production route.^[^ ^43^ ^]^ Meanwhile, transient radical capture and isotope labeling experiments confirmed this hypothesis, demonstrating that the overwhelming majority of H_2_O_2_ yield in this reaction originates from a two‐step, two‐electron O_2_ reduction pathway (Figures S23 and S24, Supporting Information).

Oxygen temperature‐programmed desorption (O_2_‐TPD) (Figure 6b) was employed to investigate the oxygen adsorption and activation capabilities of the catalysts, which correspond to the first electron transfer step of the ORR pathway (O_2_ + e^−^ → ^*^O_2_). In O_2_‐TPD analysis, the desorption peak area is proportional to the quantity of adsorbed O_2_, while the desorption temperature reflects the interaction strength.^[^ ^44^ ^]^ Notably, the KMACN‐A catalyst demonstrates both the largest O_2_ adsorption capacity and a significantly elevated desorption temperature (247 °C) compared to pristine MCN (150 °C). This indicates that the introduction of dual defects, particularly nitrogen vacancies, substantially enhances the initial adsorption and activation of molecular oxygen on the catalyst surface, effectively promoting the crucial first electron transfer to form the ^*^O_2_ radicals.

To further identify the reaction intermediates and validate the reaction pathway, in situ surface‐enhanced infrared absorption spectroscopy (SEIRAS) was employed (Figure 6c–e; Figure S25 and S26, Supporting Information). Specifically, the peaks ≈845, 1090, 1150, and 1280 cm^−1^ are assigned to surface adsorbed –COOH, C─OH, superoxide radical (∙O_2−_), and the O─O bond stretching mode in ⋅OOH, respectively.^[^ ^45^, ^46^, ^47^ ^]^ The clear observation of these key intermediates allows for a detailed deconstruction of the mechanism. The emergence of the peak at 1150 cm^−1^ (∙O_2−_, or ^*^O_2_) provides direct spectroscopic evidence for the successful execution of the first electron transfer step (O_2_ + e^−^ → *O_2_). For the high‐performance KMACN‐A catalyst, this peak exhibits a significant blueshift, signifying enhanced activation of O_2_ on its surface. Subsequently, the appearance of the band at 1280 cm^−1^ (⋅OOH) confirms that the adsorbed ^*^O_2_ intermediate is protonated in the second electron transfer step (^*^O_2_ + H⁺ + e^−^ → ^*^OOH). As illumination time increases, the intensity of the peak at 1090 cm^−1^ (C─OH) becomes significantly higher than the other spectral bands, suggesting that the C─OH groups may serve as the primary proton donors for the reaction.^[^ ^44^ ^]^ Furthermore, a quantitative analysis of the peak areas in the 3000–4000 cm^−1^ region after 60 min of reaction (Figure 6f) demonstrates that KMACN‐A yields a significantly higher concentration of H_2_O_2_. This result confirms KMACN‐A has superior surface H⁺ binding ability and high H_2_O_2_ selectivity. In summary, these findings demonstrate that enhancing the adsorption and activation of O_2_ is key to facilitating the indirect 2e^−^ ORR pathway for efficient H_2_O_2_ generation on KMACN‐A.

**Figure 7:**
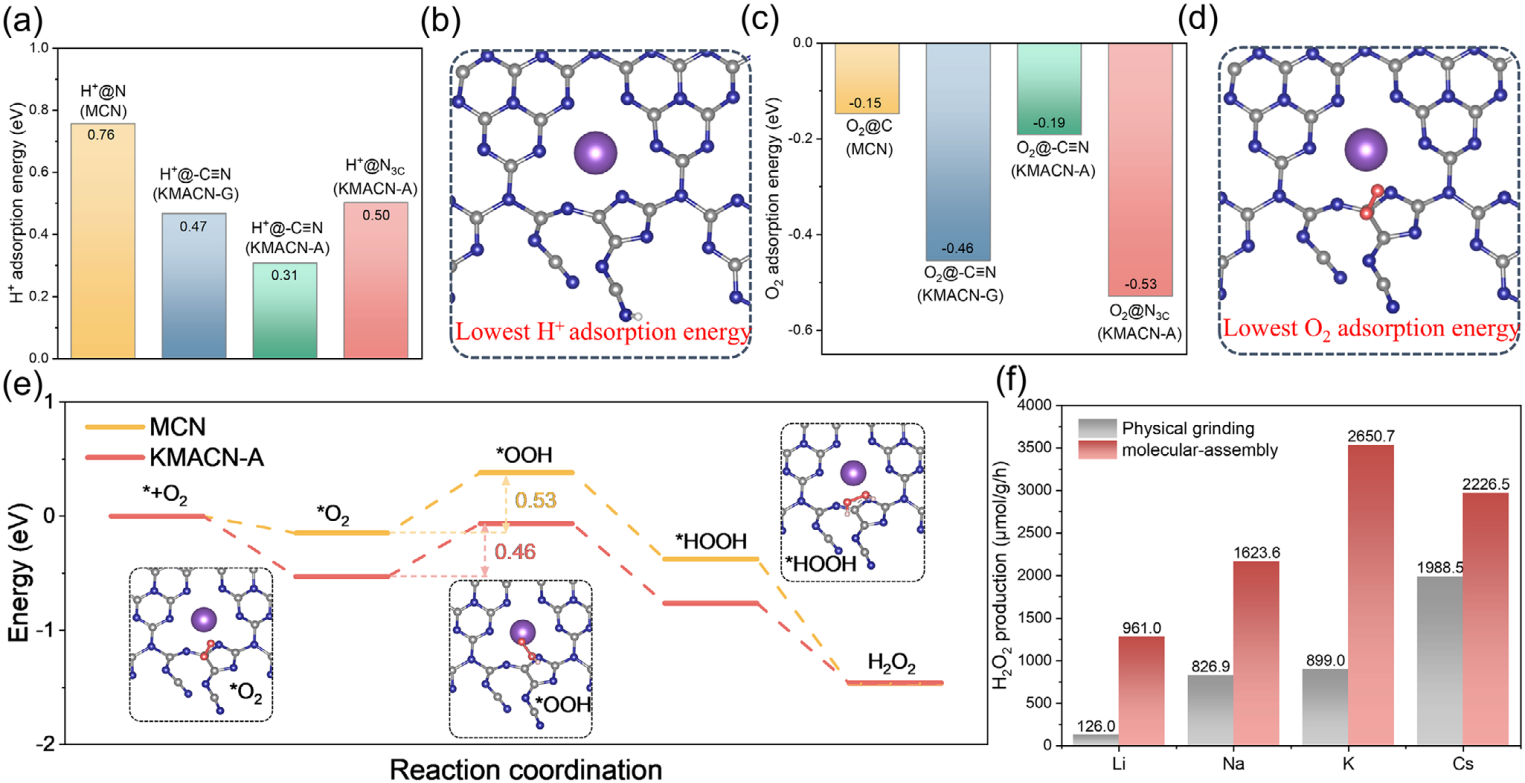
a,d) H^+^ and O_2_ adsorption energy of different sites with different models. e) Energy profiles for ORR steps on MCN and KMACN‐A. h) Photocatalytic H_2_O_2_ production by doping alkali metal elements using two different methods.

Density Functional Theory (DFT) calculations were performed to reveal the intrinsic relationship between catalyst structure and reactivity. The results indicate a strong correlation between the specific structural sites on the catalyst and its adsorption capacity for reactants. Specifically, the ‐C≡N group serves as the preferential adsorption site for H⁺, while nitrogen vacancies exhibit a stronger affinity for O_2_ (**Figure** **7**a–d; Figure S27 and S28, Supporting Information). This finding directly demonstrates that the dual‐defect structure of KMACN‐A can synergistically promote the effective adsorption and enrichment of the two key reactants, H⁺ and O_2_. To further probe the intrinsic reactivity, the energy profiles for the key electron transfer steps of the ORR process on MCN and KMACN‐A were calculated (Figure 7e). The first electron transfer step (O_2_ + e^−^ → ^*^O_2_), initiated by O_2_ adsorption, was found to be significantly promoted on the KMACN‐A surface. Meanwhile, the second electron transfer step (^*^O_2_ + H⁺ + e^−^ → ^*^OOH) was identified as the rate‐determining step (RDS) for the overall reaction. While the calculated kinetic barrier for this step on KMACN‐A is 0.46 eV, only a modest 0.07 eV lower than on MCN (0.53 eV), this value alone does not capture the full kinetic picture.^[^ ^48^ ^]^ Crucially, the enhanced exothermicity of the first electron transfer provides a strong thermodynamic driving force that also contributes to overcoming the kinetic barrier of the subsequent RDS. Therefore, the synergistic promotion – a highly favorable initial electron transfer coupled with a reduced barrier for the second – results in an overall kinetic acceleration that is substantially greater than what the 0.07 eV reduction alone would suggest.^[^ ^49^, ^50^ ^]^ This two‐fold enhancement mechanism explains the markedly superior H_2_O_2_ production activity of KMACN‐A.

Potassium, as an alkali metal element, is shown to exhibit intercalation behavior when doped into carbon nitride. The molecular assembly method ensures that K is uniformly dispersed in the precursor solution, and the subsequent calcination and polymerization processes facilitate the formation of cyano groups and nitrogen vacancies. This approach significantly enhances the catalytic activity compared to conventional physical grinding methods. To demonstrate the generalizability of this strategy, carbon nitride materials doped with other alkali metals (Li, Na, and Cs) are also prepared for photocatalytic H_2_O_2_ production (Figure 7f). As anticipated, the molecular assembly method consistently yields significantly higher activity than the conventional physical grinding method. Moreover, the FT‐IR spectra (Figure S29, Supporting Information) reveal a pronounced enhancement of the ─C≡N peak, similar to previous observations, supporting the general applicability of this doping strategy across various alkali metal elements. Thus, these findings offer a new perspective for the design of advanced catalysts and broaden the scope for future studies in this field.

## 3 Conclusion

In summary, we have demonstrated a powerful bottom‐up strategy that successfully circumvents the long‐standing synthesis conflict between creating N‐vacancies and preserving cyano groups in carbon nitride photocatalysts. By pre‐organizing precursors at the molecular level, our molecular assembly‐molten salt coupling method overcomes the inhomogeneity inherent in traditional solid‐state reactions, enabling the precise, one‐step engineering of synergistic dual‐defect active sites, achieving a H_2_O_2_ yield of 2.65 mmol·g^−1^·h^−1^. This performance is 6.2 and 3.0 times higher than that of pristine carbon nitride (MCN) and samples prepared by the physical grinding method (KMACN‐G), respectively. Systematic structural characterization and performance testing provide insight into the structure‐activity relationship of the catalyst. Structural and electronic characterization reveals the intercalation of K into the carbon nitride framework, while the supramolecular assembly strategy ensures a uniform dispersion of K within the precursor hydrogen‐bonding network. This unique preparation method ensures the precise doping of K and induces the formation of active sites, such as cyano groups and N vacancies. Theoretical calculations using Density Functional Theory (DFT) further elucidate the catalytic mechanism at an elementary level. It is revealed that the nitrogen vacancies act as powerful sites for the first electron transfer step (O_2_ → ^*^O_2_), which is significantly more exothermic on the engineered catalyst. This, in turn, provides a thermodynamic push for the subsequent rate‐determining second electron transfer step (^*^O_2_ → ^*^OOH), whose kinetic barrier is also lowered by the synergistic dual‐defect structure. In situ ATR‐SEIRAS and radical trapping experiments corroborate this two‐step, one‐electron ORR pathway. This work not only provides an efficient photocatalyst for H2O2 production but, more importantly, establishes a new and generalizable paradigm for the rational design of advanced functional materials. This bottom‐up approach, which allows for precise control over dopant distribution and defect formation, holds significant promise for engineering a wide range of other 2D materials for diverse applications in catalysis, energy, and beyond.

## 4 Experimental Section

### Chemicals

Melamine (C_3_H_6_N_6_), cyanuric acid (C_3_H_3_N_3_O_3_), potassium chloride (KCl), sodium sulfate (Na_2_SO_4_), and Nafion solution (5 wt.%) were purchased from Macklin Biochemical Technology Co., Ltd. (Shanghai, China). Potassium iodide (KI) was obtained from Energy Chemical (Shanghai, China). Potassium hydrogen phthalate (C_8_H_5_KO_4_), potassium hydroxide (KOH), and ethanol (C_2_H_5_OH) were supplied by Aladdin Biochemical Technology Co., Ltd. (Shanghai, China). Ammonium fluoride (NH_4_F, ≥ 96%), hydrochloric acid (HCl, 36%), and lactic acid (C_3_H_6_O_3_) were procured from Sinopharm Chemical Reagent Co., Ltd. All chemicals and reagents were of analytical grade and used as received without further purification. All aqueous solutions used in the experiments were prepared using ultrapure water (Millipore, 18.25 MΩ·cm).

### Synthesis of KMACN‐A

The KMACN‐A was synthesized via a molecular assembly‐molten salt coupling strategy through a two‐step polymerization process. In this procedure, 2.5 g of melamine, 2.5 g of cyanuric acid, and 5.0 g of KCl (as the potassium source) were thoroughly ground and homogenized. The mixture was then dispersed in deionized water and stirred at 80 °C in a water bath for several hours, followed by overnight drying. The dried mixture was transferred into a covered crucible and calcined at 550 °C in a Muffle furnace for 2 h with a heating rate of 5 °C min^−1^. After cooling to room‐temperature, the obtained powders were washed with hot water to remove unreacted salts several times and vacuum dry at 80 °C for 12 h.

### Synthesis of KMACN‐G

The KMACN‐G was prepared via a physical mixing method using the same precursors as KMACN‐A. After uniform grinding, the mixture was directly transferred into a covered crucible and calcined at 550 °C in a Muffle furnace for 2 h with a heating rate of 5 °C min^−1^. After cooling to room‐temperature, the obtained powders were washed with hot water to remove unreacted salts several times and vacuum dry at 80 °C for 12 h.

### Synthesis of KMCN‐G

The KMCN‐G was synthesized according to the previous work,^[^ ^8^ ^]^ which was identical to the preparation of KMACN‐G except that cyanuric acid was excluded. Instead, a total of 5 g of melamine was used as the sole precursor except for KCl.

### Synthesis of MACN

The MACN was prepared via the self‐assembly method under the same conditions as KMACN‐A, except that no KCl was added during the polymerization process.

### Synthesis of MCN

The MCN was synthesized via a conventional thermal pyrolysis method. Briefly, 10 g of melamine was calcined in a muffle furnace at 550 °C in a Muffle furnace for 2 h with a heating rate of 5 °C min^−1^. After cooling to room‐temperature, the obtained powders were washed with deionized water several times and vacuum dried at 80 °C for 12 h.

### Photocatalytic Tests

The photocatalytic synthesis of hydrogen peroxide experiment was conducted in a 100 mL external irradiation photoreactor at room‐temperature and pressure. A 300 W Xe lamp with λ ≥ 420 nm filter was used as the radiation source. Typically, 80 mg of photocatalyst was added to 80 mL of an aqueous solution containing 10 vol.% lactic acid as a sacrificial agent under magnetic stirring. Before irradiation, the photocatalytic system was purged through O_2_ (purity ≥ 99.9%) for 20 min in the dark to achieve adsorption–desorption equilibrium. The whole reaction was controlled at a constant temperature of 20 °C under the action of a circulating chiller, and the reaction time was 1 h. In this experiment, 1.5 mL of solution was extracted every 15 min and filtered through a 0.22 µm polytetrafluoroethylene filter head to remove the photocatalyst. The concentration of hydrogen peroxide was calculated by measuring the absorbance at 350 nm with a UV–vis spectrophotometer shown in Figure S1 (Supporting Information).

### Characterization

The morphology and microstructure of the samples were investigated using scanning electron microscopy (SEM) images obtained from the Nova NanoSEM 450 field emission scanning electron microscope and transmission electron microscopy (TEM) images acquired through the Talos F200X field emission transmission electron microscope. Spherical aberration transmission electron microscopy (AC‐TEM) using JEM‐ARM300F. X‐ray diffraction (XRD) patterns were collected using a Bruker diffractometer under Cu Kα radiation (λ = 1.540589 Å) within a 2θ range of 10–80°, with a step size of 0.12° and a scanning speed of 10° min^−1^. Fourier‐transform infrared (FT‐IR) spectra were recorded using the PerkinElmer Spectrum 100 spectrometer (Thermo Fisher, USA). The samples were dried and mixed with KBr, followed by tablet formation for measurement. Raman spectroscopy was performed under ambient conditions using a LabRAM HR micro‐confocal Raman spectrometer (Horiba J.Y.) with a 785 nm laser source. X‐ray photoelectron spectroscopy (XPS) was conducted using a Thermo Scientific K‐Alpha spectrometer, with all binding energy values calibrated to the C 1s signal at 284.8 eV. Sputter etching was performed using an Ar^+^ ion gun on a 2 × 2 mm raster size, with an ion energy set to 2000 eV. Two etching levels were conducted, each lasting 50 s, and spectra were obtained after each etching step. UV–vis diffuse reflectance spectra were recorded using a UV‐2700 UV–vis spectrophotometer (Shimadzu, Japan). Electron paramagnetic resonance (EPR) spectroscopy was measured using a Bruker EMXplus‐6/1 electron paramagnetic resonance spectrometer, with a 300 W visible light xenon lamp used to irradiate the catalyst, measuring the EPR signal intensity in both dark and illuminated conditions over 120 s. Thermogravimetric analysis (TG) was conducted on a Netzsch thermal analyzer, heating the sample from room‐temperature to 800 °C at a rate of 20 °C min^−1^ under an Ar atmosphere. Oxygen temperature‐programmed reduction (O_2_‐TPD) experiments were carried out using a VDSorb‐91i‐VAP‐HB‐MS fully automated programmed temperature chemical adsorption instrument, with a mass spectrometer recording the O_2_ signal. Before O_2_ adsorption, 50 mg of the catalyst was heated to 200 °C under He (50 mL min^−1^) at a rate of 10 °C min^−1^ and purged for 1 h to remove surface‐adsorbed water and oxygen. After cooling to 25 °C, the flow gas was switched to 99.9% O_2_ (30 mL min^−1^) for 1 h of adsorption at room‐temperature. The He gas was then cut off, and the catalyst was heated to 550 °C at a rate of 10 °C min^−1^ to investigate its oxygen adsorption capacity.

### Photocurrent Measurement

Photocurrent measurements, including transient photocurrent response(I‐T), electrochemical impedance spectroscopy (EIS), and Mott–Schottky analysis, were performed at room‐temperature using a three‐electrode system on a (CORRTEST CS310H) electrochemical system with a standard three electrode system included a counter electrode (Pt wire), working electrode and reference electrode (Ag/AgCl), and a 0.5 m Na_2_SO_4_ aqueous solution as the supporting electrolyte. The catalyst suspension was uniformly drop‐coated onto ITO glass (1 × 1 cm^2^) using a simple pipetting method and subsequently dried in an oven at 60 °C to fabricate the working electrode, and the light source was a 300 W Xe lamp.

### In situ ATR‐SEIRAS

In situ Attenuated Total Reflection‐Surface Enhanced Infrared Absorption Spectroscopy (In situ ATR‐SEIRAS) measurements were conducted on the PerkinElmer Spectrum 100 spectrometer, equipped with a mercury cadmium telluride (MCT) detector, a variable angle reflection accessory (provided by Jiaxing Puxiang Technology Co., Ltd.), and a battery (also provided by Jiaxing Puxiang Technology Co., Ltd.). Before photocatalytic measurements, the reaction liquid was injected, and a 20% O_2_/Ar gas mixture was introduced for 40 min under dark conditions to achieve adsorption equilibrium. Subsequently, photoreaction was conducted, with the light source irradiating the catalyst surface from above, and spectra were collected every 5 min for 60 min while continuously introducing O_2_ to facilitate the photocatalytic reaction.

### Computational Details

First‐principles DFT calculations were performed using the Vienna ab initio simulation package (VASP) and the projector augmented wave (PAW) method. The exchange‐correlation effects were treated in generalized gradient approximation (GGA) with the Perdew–Burke–Ernzerhof (PBE) potential. The kinetic energy cutoff was chosen to be 400 eV. The electronic energy was considered self‐consistent when the energy change was smaller than 10^−5^ eV. A geometry optimization was considered convergent when the force change was smaller than 0.02 eV Å^−1^. Grimme's DFT‐D3 methodology was used to describe the dispersion interactions.

The MCN single‐layer unit cells were placed into a vacuum at a depth of 15 Å, and the Brillouin zone was sampled using a 4 × 4 × 1 gamma k‐point grid. Slab model was constructed in a 3 × 2 supercell, with a vacuum layer of 15 Å in the z direction to avoid the interaction between layers. To simulate K‐doped CN, we add K and defect groups into the supercell of MCN, and then perform simulation calculations under the same conditions of other parameters. During structural optimization, all atoms are allowed to relax.

The adsorption energy (Eads) was defined as the following equation

(2) $$E_{ads}=E_{ad/sub}\;-E_{ad}-E_{sub}$$

where *E_ad/sub_*, *E_ad_*, and *E_sub_* are the total energies of the optimized adsorbate/substrate system, the adsorbate in the gas phase, and the clean substrate, respectively. The Brillouin zone was sampled using a 1 × 1 × 1 Gamma point grid for optimization of gas molecules.

## Conflict of Interest

The authors declare no conflict of interest.

## Supporting information



Supporting Information

## Data Availability

The data that support the findings of this study are available from the corresponding author upon reasonable request.
