# Supplementary material for: Molecular Assembly Unlocks Dual‐Defect Synergy in Carbon Nitride for Efficient H2O2 Photosynthesis
Source: Adv Sci (Weinh). 2025 Nov 8;13(5):e17957. doi: 10.1002/advs.202517957 (PMC12850089; doi:10.1002/advs.202517957)
Supplement: Supplementary file 1 — Supporting Information [file ADVS-13-e17957-s001.pdf]

Supporting Information

Molecular Assembly Unlocks Dual-Defect Synergy in Carbon Nitride for Efficient H_2O_2 Photosynthesis

Xiaolin Sun[a], Pengfei Tian[b], Jinye Li[a], Minghui Zhu[a]*, Jing Xu[a,c]*, Fu-Zhen Xuan[b,]*

[a] *State Key Laboratory of Green Chemical Engineering and Industrial Catalysis, School of Chemical Engineering, East China University of Science and Technology, Shanghai 200237, China*

[b] *Key Laboratory of Pressure Systems and Safety (Ministry of Education), School of Mechanical and Power Engineering, East China University of Science and Technology, Shanghai 200237, China*

[c] *University Engineering Research Center of Green Chemical New Materials, School of Chemistry and Chemical Engineering, Guangxi University, Nanning, Guangxi, 530004, PR. China.*

Corresponding authors:

minghuizhu@ecust.edu.cn; xujing@ecust.edu.cn; fzxuan@ecust.edu.cn

Contents

Figure S1. The working curve of concentration of H_2O_2 and absorption. 5

Figure S2. The SEM image of all samples. 6

Figure S3. a-b) TEM micrograph, c) HRTEM images of KMACN-G. d) EDS elemental mapping of KMACN-G. 7

Figure S4. AC-TEM micrograph of a) KMACN-A and b) KMACN-G. 8

Figure S5. The 5mM H_2O_2 decomposition experiment of all samples (under reaction conditions). 9

Figure S6. Theoretical calculations of inserting K atoms between the layers of carbon nitride. 10

Figure S7. FTIR spectra of MACN. 11

Figure S8. Cyclic experiments over 4 cycles for H_2O_2 activity using the KMACN-A. 12

Figure S9. a) XRD pattern and b) FT-IR spectrum of fresh KMACN-A and used after the photocatalytic reaction. 13

Figure S10. TEM micrograph, HRTEM images, and EDS elemental mapping of a) KMACN-A and b) KMACN-G after the photocatalytic reaction. 14

Figure S11. The color of catalyst KMACN-A, a) before and b) after the reaction. 15

Figure S12. a) XRD pattern and b) FT-IR spectrum of fresh KMCN-G and used after the photocatalytic reaction. 16

Figure S13. a) XRD pattern and b) FT-IR spectrum of fresh KMACN-G and used after the photocatalytic reaction. 17

Figure S14. The high-resolution XPS spectra for MACN. 18

Figure S15. The high-resolution XPS spectra of O 1s for the MCN and various K-CN. 19

Figure S16. ESR spectra of a) KMCN-G and b) KMACN-G under dark and light conditions. 20

Figure S17. DTG profile for different photocatalysts measured in Ar atmosphere at a flow rate of 20 mL/min. 21

Figure S18. Mott-Schottky plots of (a) MACN, (b) KMCN-G, and (c) KMACN-A in 0.5 M Na_2SO_4. 22

Figure S19. Valence band of MACN, KMCN-G, and KMACN-A from XPS spectra. 23

Figure S20. (a) three types of N_{3C} positions selected on KMACN-G. (b-d) three kinds of NV-x (x=1, 2, 3) with different relative positions of -C≡N group and N_{3C} vacancy after structural optimization. (e) The formation energy of N_{3C} vacancies in different positions (including original data). (The blue, gray, and purple spheres are represented by N, C, and K atoms, respectively.). 24

Figure S21. The structure and calculated density of states (DOS) of KMACN-G. 26

Figure S22. equivalent circuit modeling of the electrochemical impedance spectroscopy (EIS) data. 27

Figure S23. DMPO-EPR radical trapping experiment for a) superoxide anion radical and b) hydroxyl radical of KMACN-A. 28

Figure S24. Kinetic Isotope Effect (KIE) experiment in different atmospheres. 29

Figure S25. Schematic diagram of in-situ ATR SEIRAS spectra of photocatalysis of H_2O_2 from O_2 by CN. 30

Figure S26. In-situ ATR SEIRAS spectra of photocatalysis of H_2O_2 from O_2 by a) and b) KMCN-G. 31

Figure S27. The position of H^+ adsorption and the corresponding H^+ adsorption energy on g-C_3N_4 models with different defect structures. The white spheres represent the adsorbed H^+. 32

Figure S28. The position of O_2 adsorption and the corresponding O_2 adsorption energy on g-C_3N_4 models with different defect structures. 33

Figure S29. FT-IR spectrum of different elements CN catalysts (a)Li, (b)Na, and (c)Cs. prepared by different methods, physical grinding (black line); molecular assembly-molten salt coupling (red line) 34

Table S1. A literature survey for the production rate of H_2O_2 by different catalysts. 35

Table S2. FWHM values of (002) crystal planes in Figure 2a. 36

Table S3. XPS surface atoms ratio and N/C. 37

Table S4. XPS surface total atom ratio 38

Table S5. The fitted parameters in EIS. 39

References 40

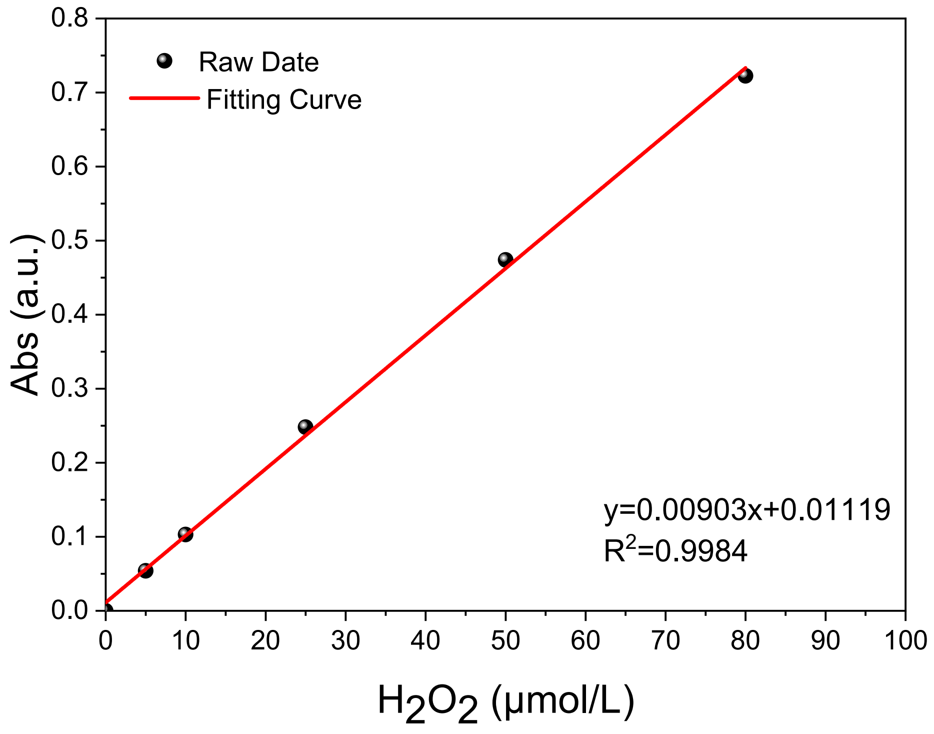

Figure S1. The working curve of concentration of H_2O_2 and absorption.

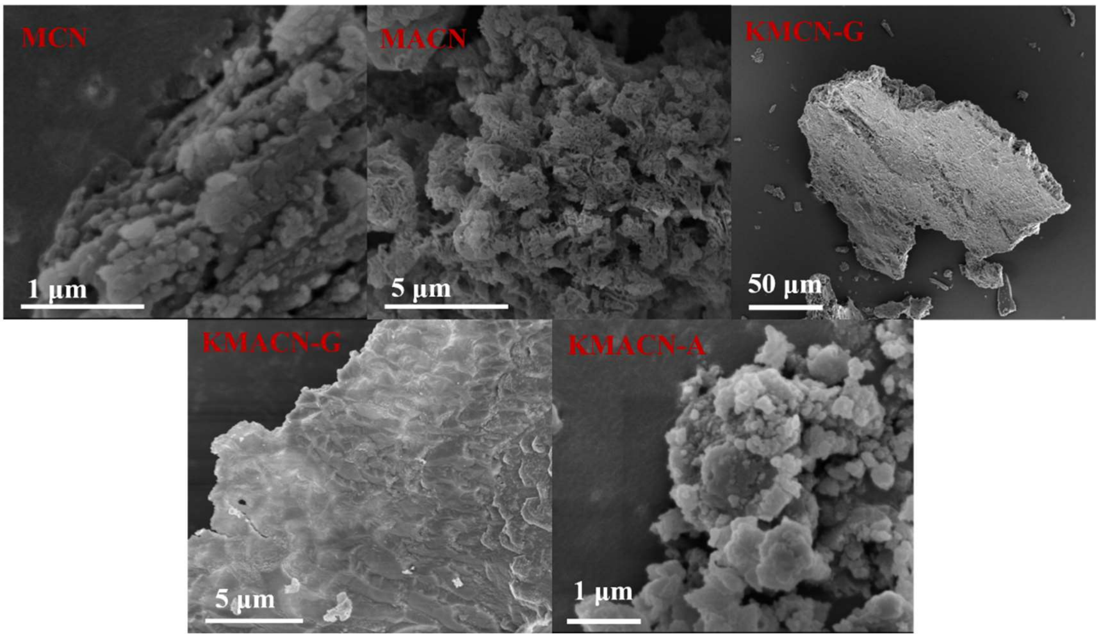

Figure S2. The SEM image of all samples.

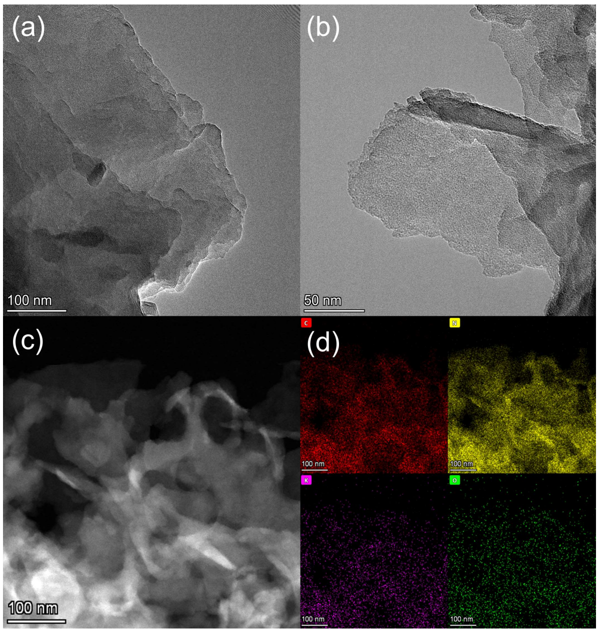

Figure S3. a-b) TEM micrograph, c) HRTEM images of KMACN-G. d) EDS elemental mapping of KMACN-G.

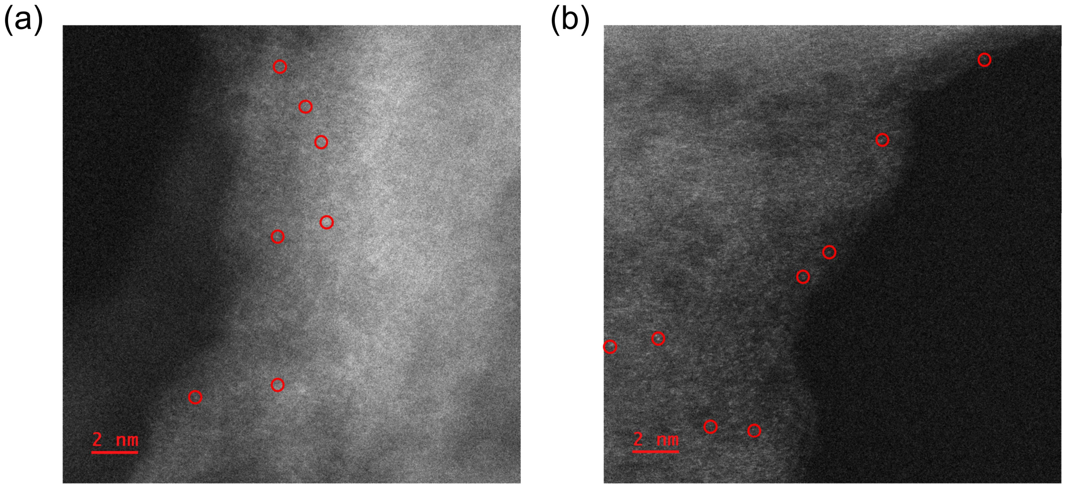

Figure S4. AC-TEM micrograph of a) KMACN-A and b) KMACN-G.

We captured high-magnification aberration-corrected electron microscopy images[1] revealing K atoms dispersed within the layered structure of carbon nitride, appearing as brighter atomic dots.

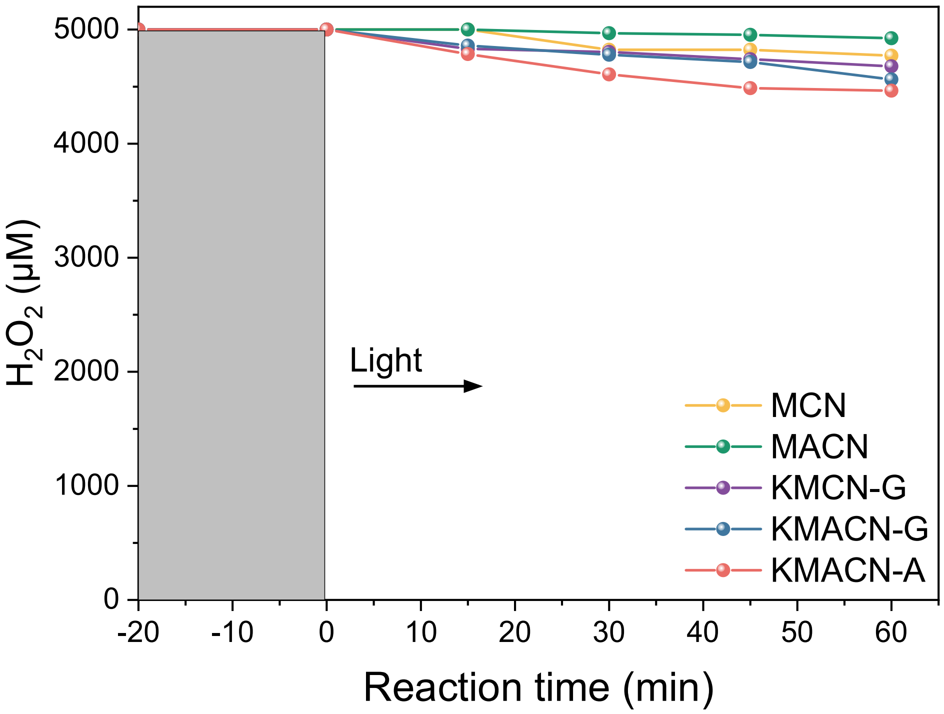

Figure S5. The 5mM H_2O_2 decomposition experiment of all samples (under reaction conditions).

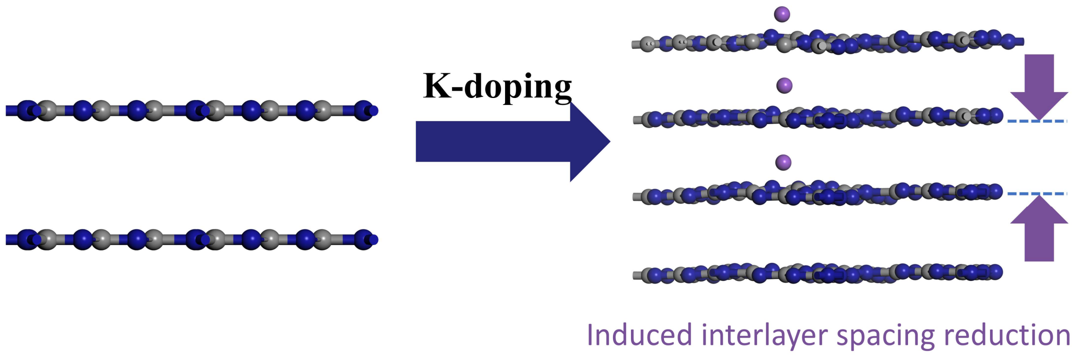

Figure S6. Theoretical calculations of inserting K atoms between the layers of carbon nitride.

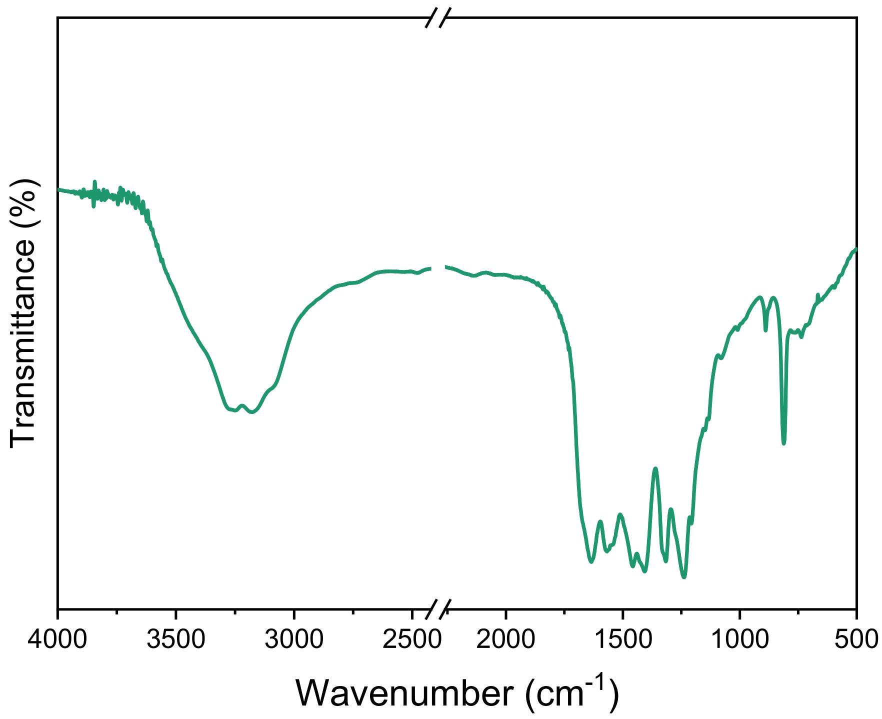

Figure S7. FTIR spectra of MACN.

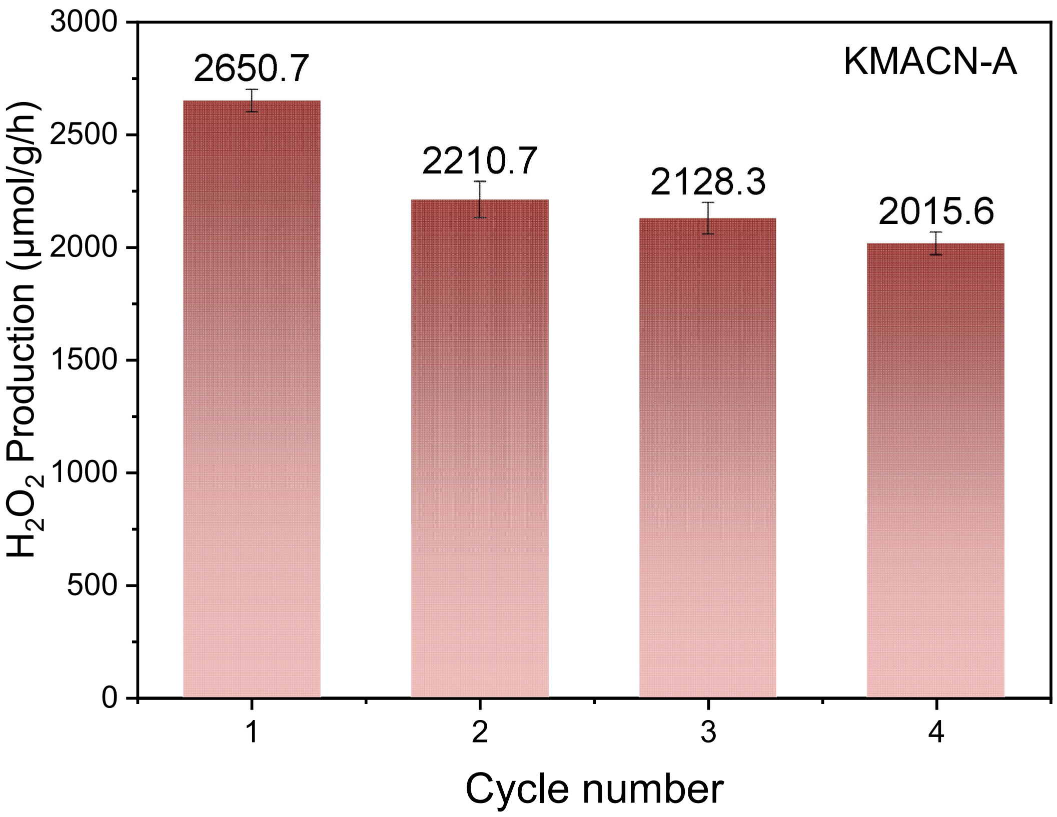

Figure S8. Cyclic experiments over 4 cycles for H_2O_2 activity using the KMACN-A.

The stability of the KMACN-A photocatalyst was evaluated through four cycles of H_2O_2 photosynthesis. After four cycles of 60 minutes per photocatalytic reaction, the H_2O_2 synthesis rate was 77% of that of the original photocatalyst (Figure S7).

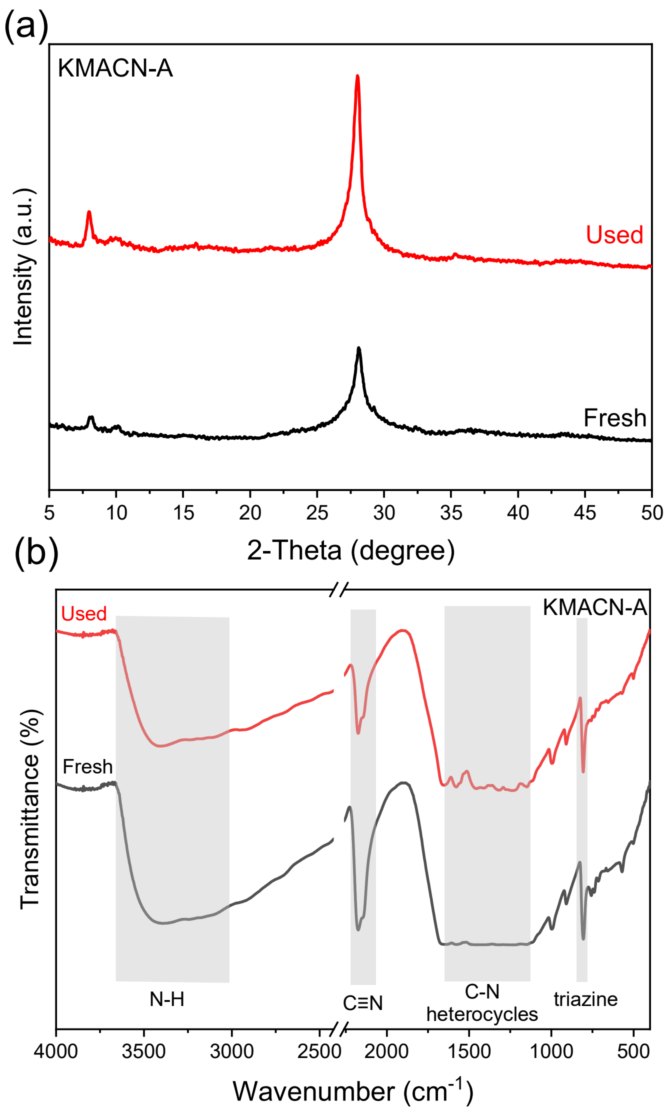

Figure S9. a) XRD pattern and b) FT-IR spectrum of fresh KMACN-A and used after the photocatalytic reaction.

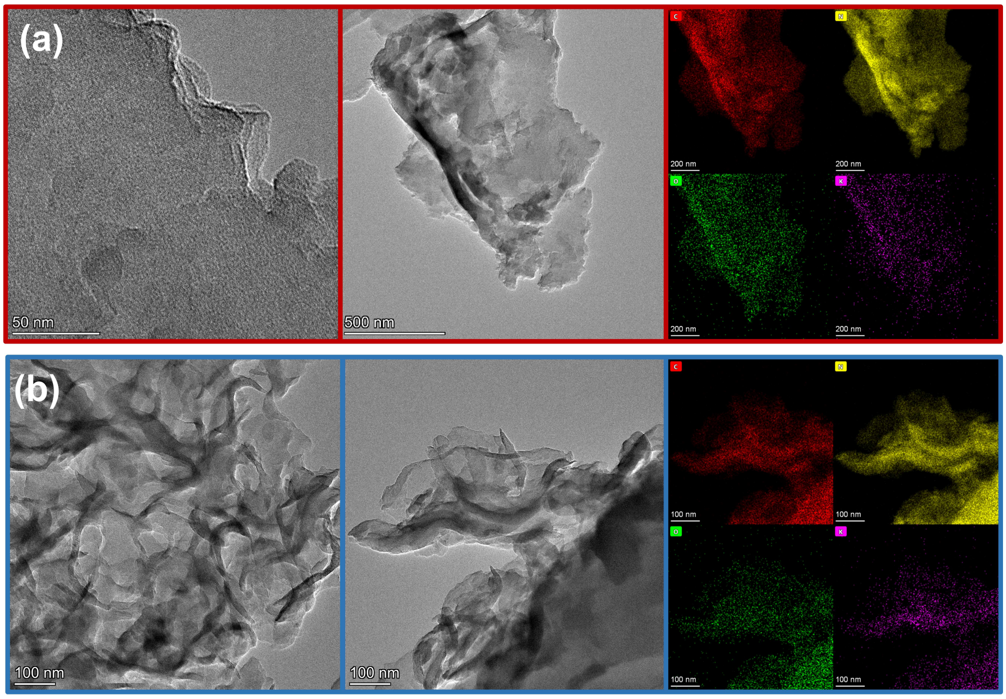

Figure S10. TEM micrograph, HRTEM images, and EDS elemental mapping of a) KMACN-A and b) KMACN-G after the photocatalytic reaction.

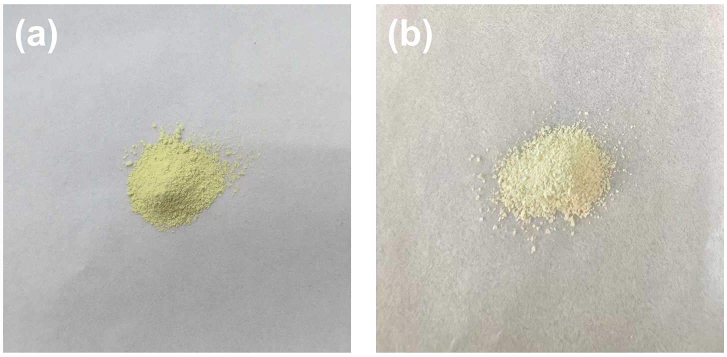

Figure S11. The color of catalyst KMACN-A, a) before and b) after the reaction.

The XRD, TEM, and FT-IR of the reacted KMACN-A were then tested (Figure S8–9). The XRD phase structure remained unchanged, indicating that the catalyst's original structure was retained after the reaction. The HRTEM still showed a layered structure, and the elements in the mapping diagram were uniformly distributed without agglomeration. However, the FT-IR spectra showed that the peak at 2172 cm^{-1} formed by the -C≡N group weakened after the reaction, implying that the -C≡N group may have been destroyed during the reaction[2]. The samples changed from dark yellow to yellowish-white, indicating decreased light absorbance after the reaction (Figure S10)[3]. The active sites may have been attacked by hot electrons, hot holes, and reactive oxygen species (e.g., hydroxyl radicals). Additionally, mass loss through centrifugation after each cycle can lead to decreased photocatalytic activity. Furthermore, the XRD and FT-IR of the two catalysts, KMACN-G and KMCN-G, were measured after the reaction, as illustrated in Figures S11–S12. This confirmed that the above catalysts exhibited a certain level of destruction of the cyanide active sites after the reaction.

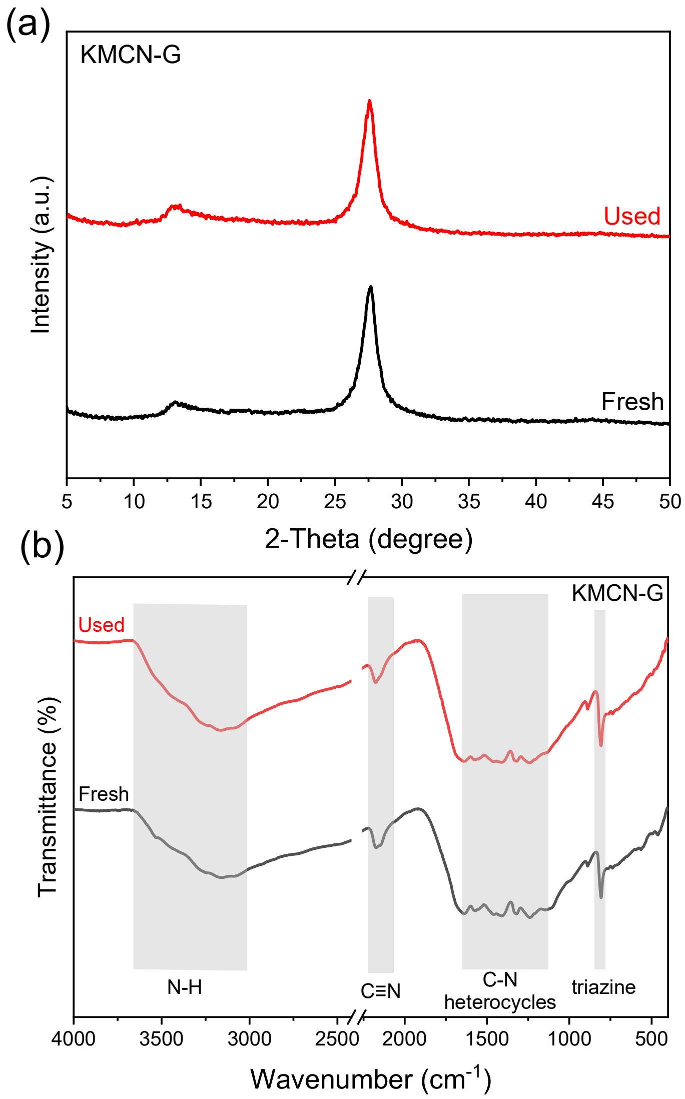

Figure S12. a) XRD pattern and b) FT-IR spectrum of fresh KMCN-G and used after the photocatalytic reaction.

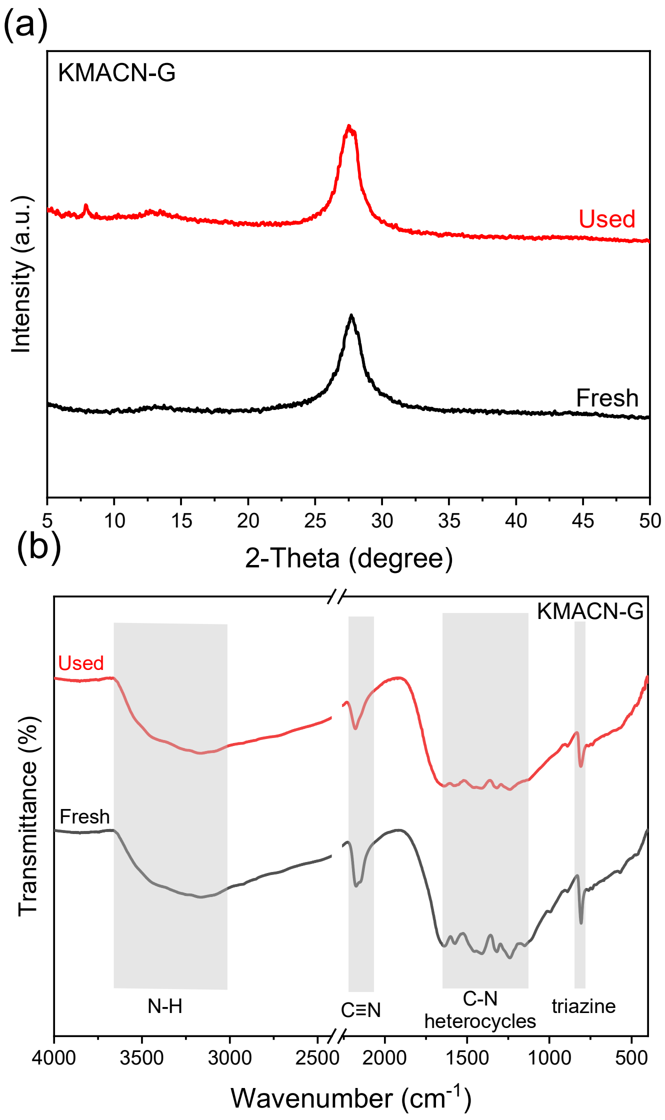

Figure S13. a) XRD pattern and b) FT-IR spectrum of fresh KMACN-G and used after the photocatalytic reaction.

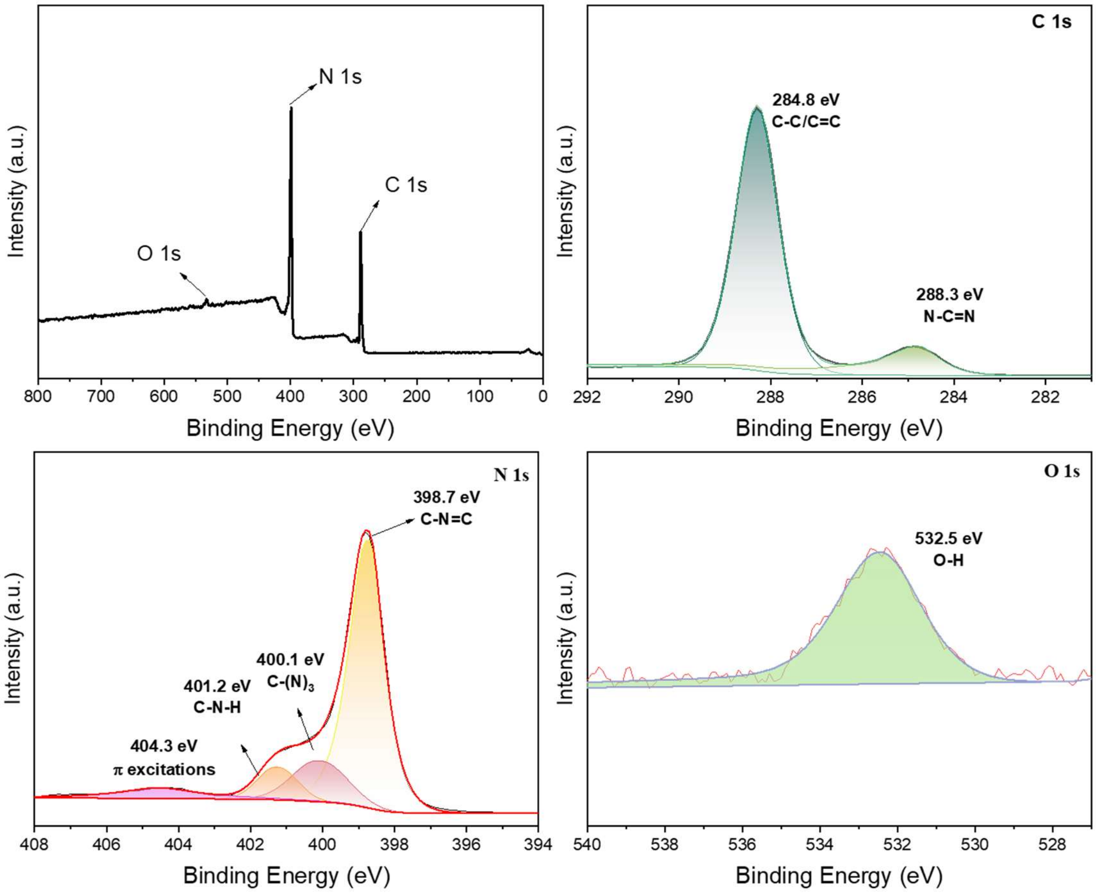

Figure S14. The high-resolution XPS spectra for MACN.

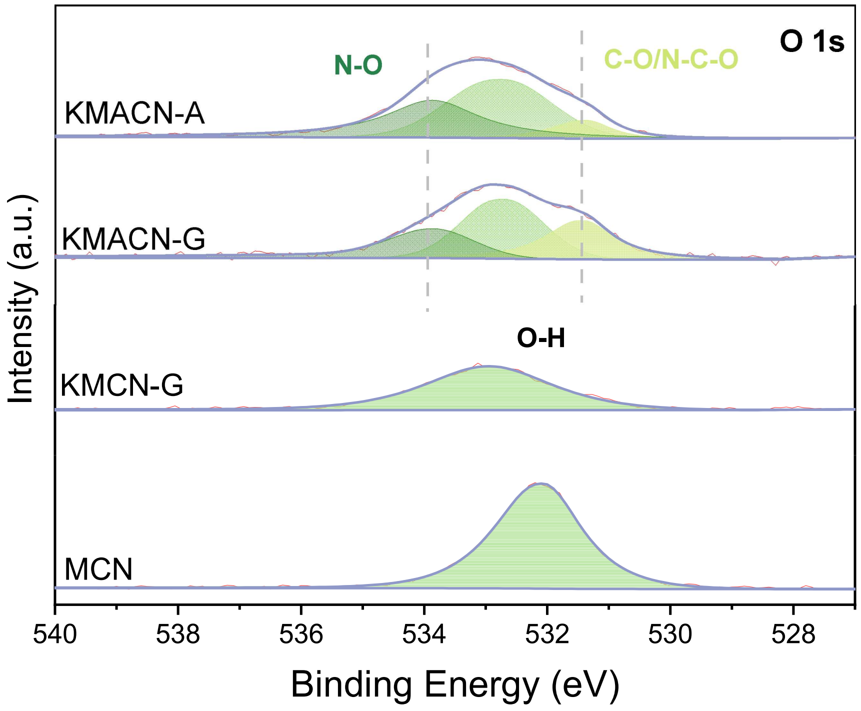

Figure S15. The high-resolution XPS spectra of O 1s for the MCN and various K-CN.

In the O 1s spectrum (Figure S7), besides the peaks attributed to adsorbed water (533.4 eV) and intermediate O-H species formed during thermal polymerization (532.1 eV) [4], a new peak at 530.7 eV emerges in both KMACN-G and KMACN-A, which is assigned to N-O/N-C-O species, possibly due to the partial substitution of protons by alkali metal cations [5]

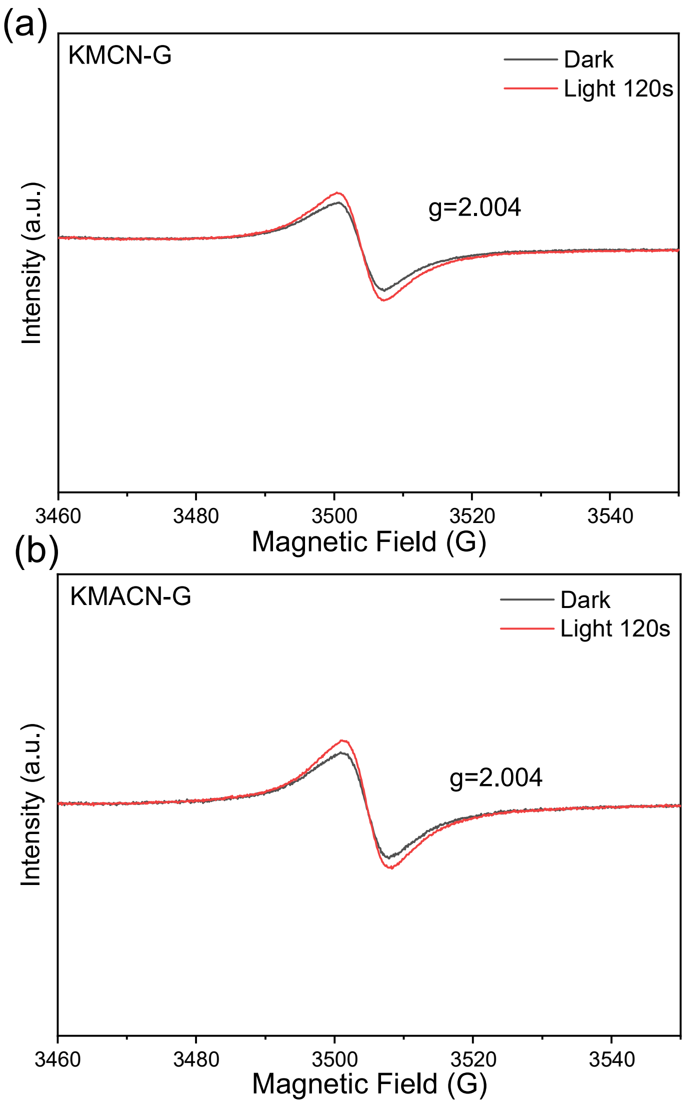

Figure S16. ESR spectra of a) KMCN-G and b) KMACN-G under dark and light conditions.

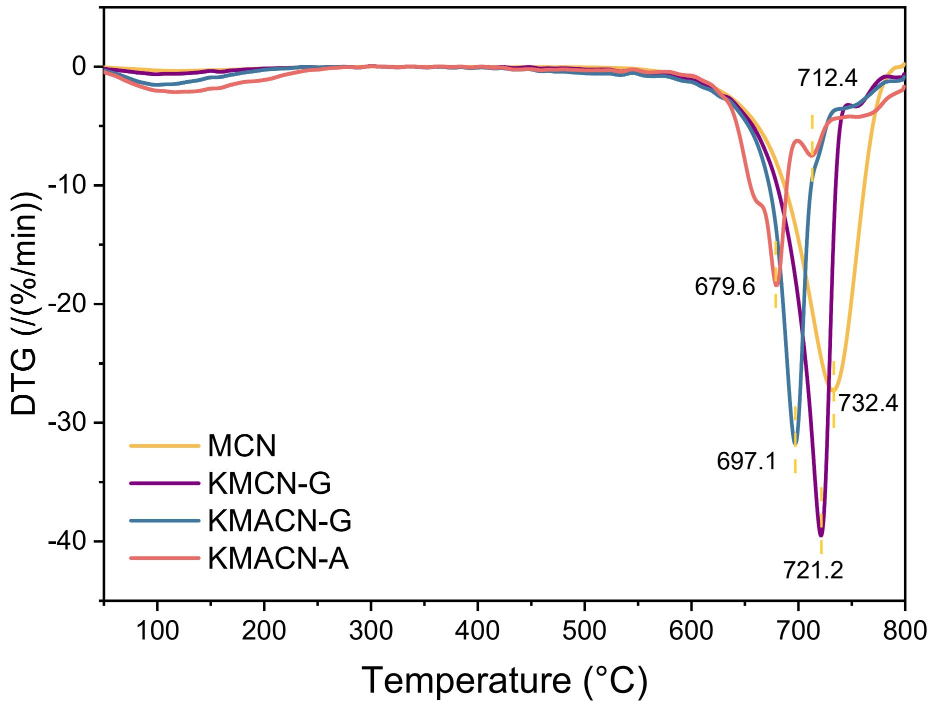

Figure S17. DTG profile for different photocatalysts measured in Ar atmosphere at a flow rate of 20 mL/min.

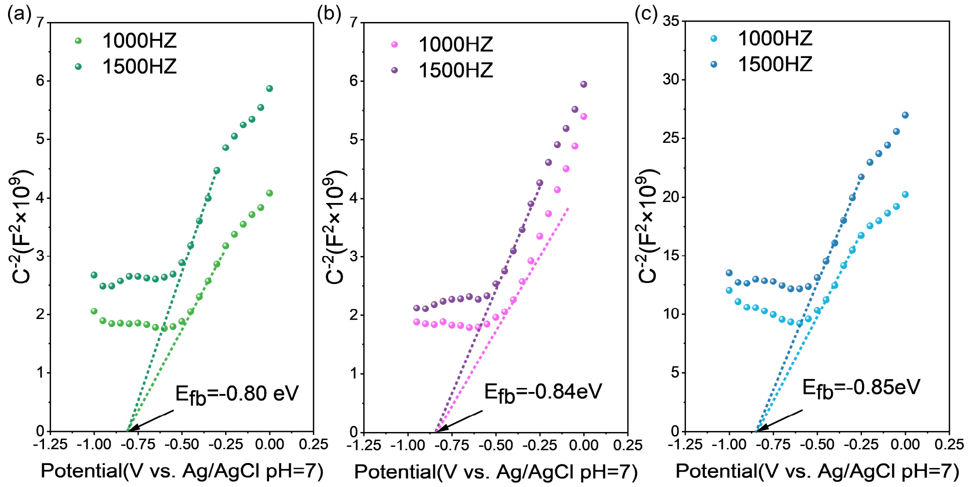

Figure S18. Mott-Schottky plots of (a) MACN, (b) KMCN-G, and (c) KMACN-A in 0.5 M Na_2SO_4.

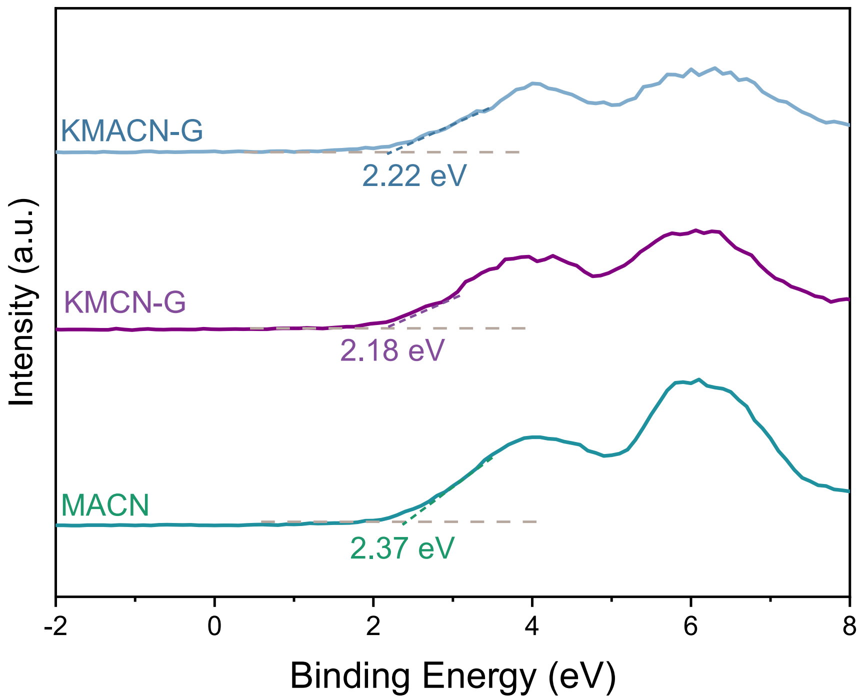

Figure S19. Valence band of MACN, KMCN-G, and KMACN-A from XPS spectra.

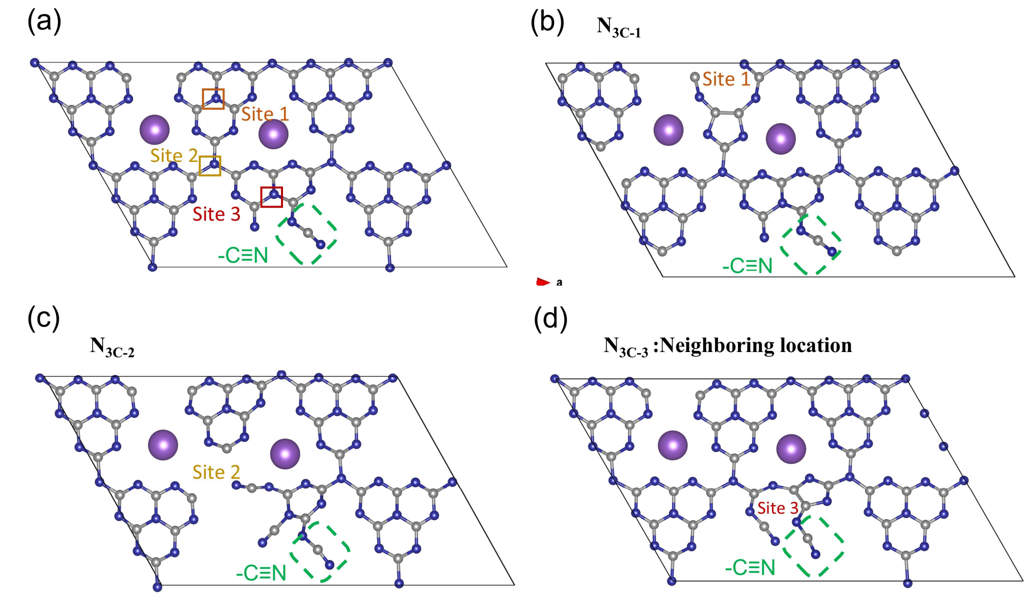

(e)

Location of N_{3C}	N_{3C} vacancy formation energy	$E_{(N3C)}$	$E_{(N\ atom)}$	$E_{(a)}$
1	6.56	-696.39	-3.12	-706.07
2	6.90	-696.06	-3.12	-706.07
**3**	**5.15**	-697.81	-3.12	-706.07

Figure S20. (a) three types of N_{3C} positions selected on KMACN-G. (b-d) three kinds of NV-x (x=1, 2, 3) with different relative positions of -C≡N group and N_{3C} vacancy after structural optimization. (e) The formation energy of N_{3C} vacancies in different positions (including original data). (The blue, gray, and purple spheres are represented by N, C, and K atoms, respectively.).

Figure S19a illustrates three selected N_{3C} atoms with -C≡N groups in different positions within KMACN-G. Subsequently, these three N_{3C} vacancies in KMACN-G were created sequentially, and the formation energies of these N_{3C} vacancies were calculated (Figure S19e). Figures S2b–d show the three structurally optimized models as double defect sites of g-C_3N_4 with different positions of the -C≡N group relative to the N vacancy and these models are named N_{3C-x} (x = 1, 2, 3). The computational results and

raw data are shown in Figure S19e. Among these models, N_{3C-3} has the lowest N_{3C} vacancy formation energy, corresponding to the nearest neighbor position of the C≡N group and the N_{3C} vacancy. Based on the principle of energy minimization, the -C≡N group is more likely to form with the nearest N_{3C} vacancy, suggesting that this neighboring position is more stable. Thus, we determined the N vacancy site and structure in KMACN-A (Figure S19d).

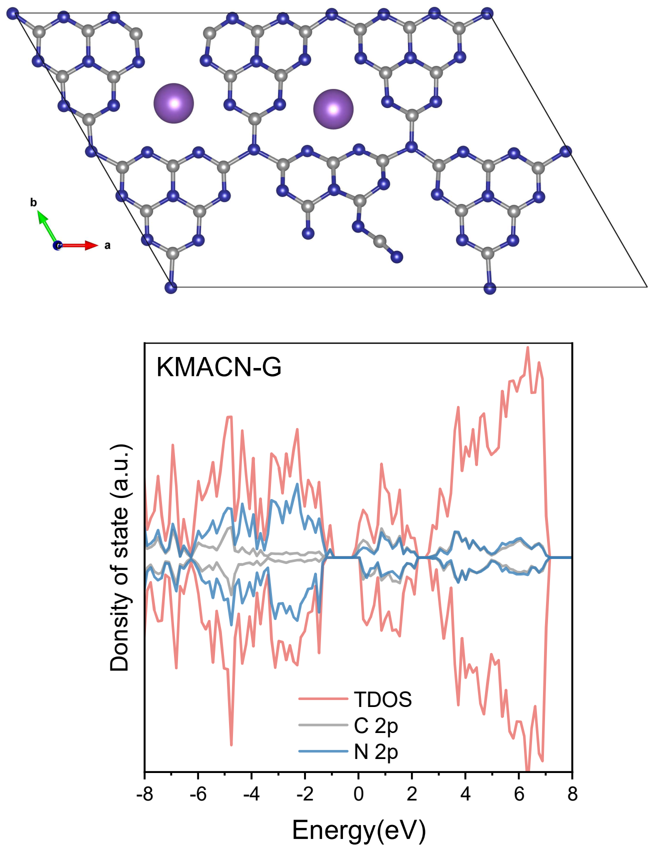

Figure S21. The structure and calculated density of states (DOS) of KMACN-G.

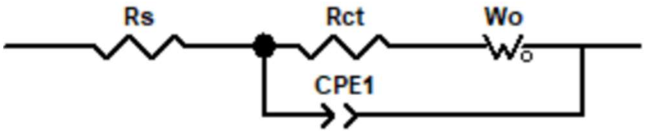

Figure S22. equivalent circuit modeling of the electrochemical impedance spectroscopy (EIS) data.

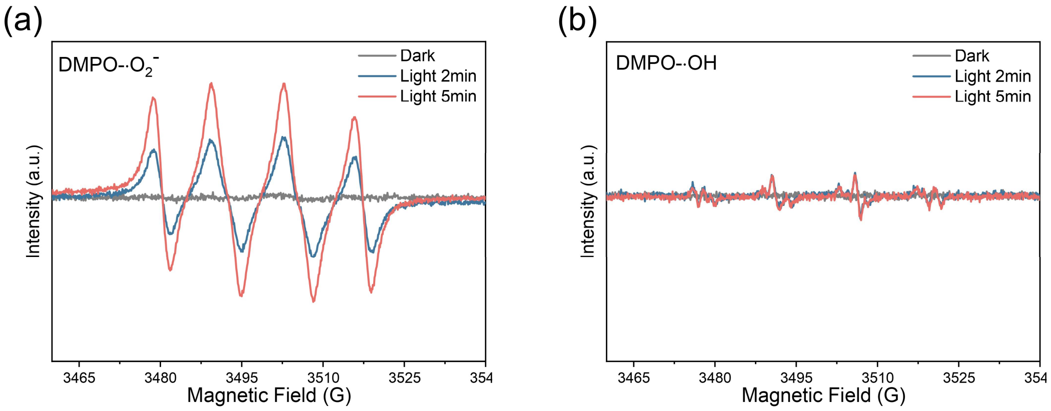

Figure S23. DMPO-EPR radical trapping experiment for a) superoxide anion radical and b) hydroxyl radical of KMACN-A.

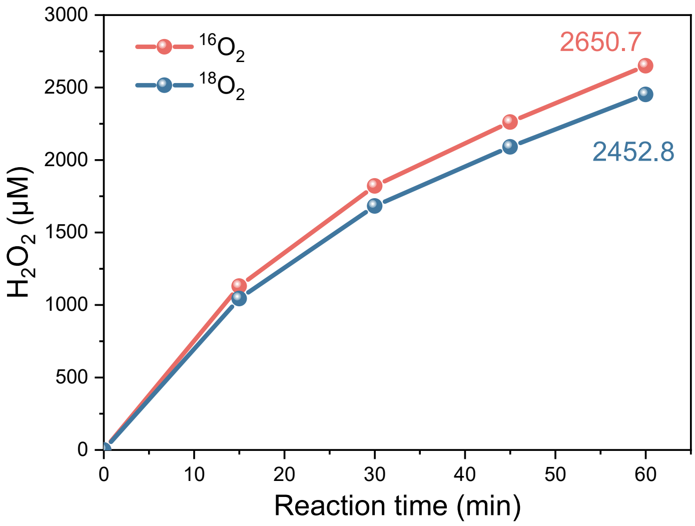

Figure S24. Kinetic Isotope Effect (KIE) experiment in different atmospheres.

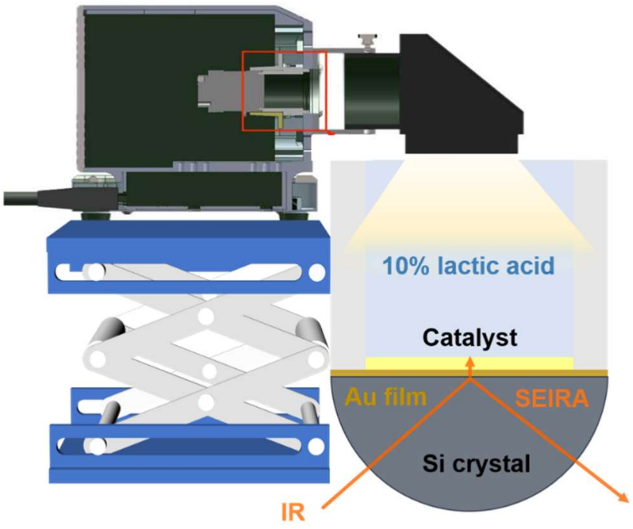

Figure S25. Schematic diagram of in-situ ATR SEIRAS spectra of photocatalysis of H_2O_2 from O_2 by CN.

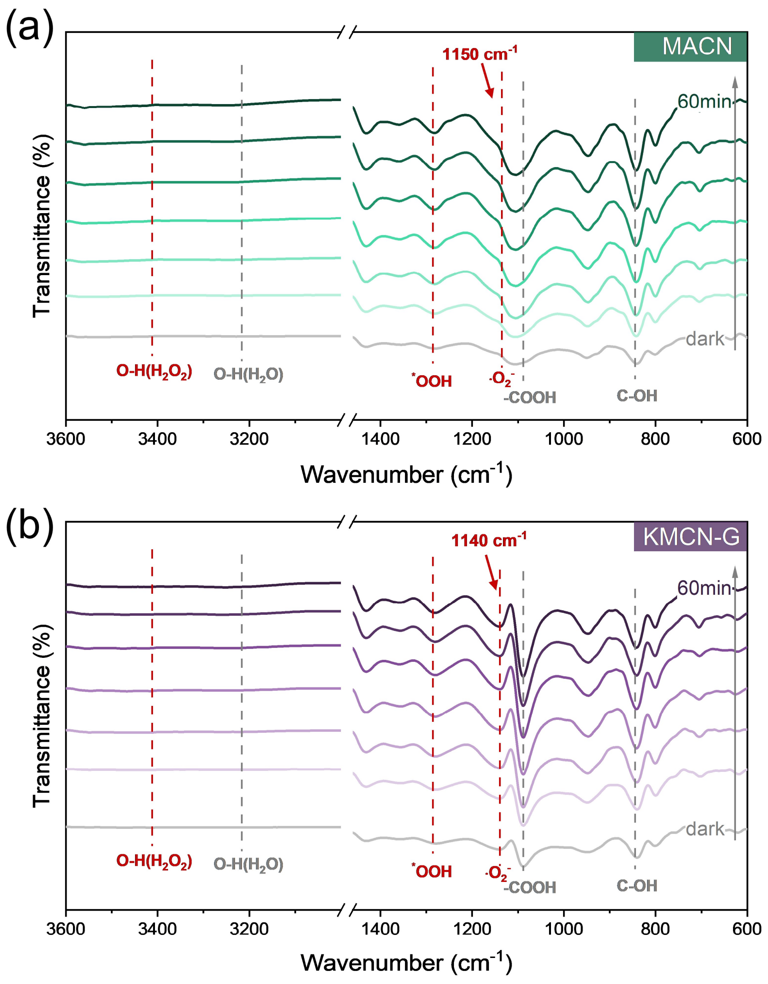

Figure S26. In-situ ATR SEIRAS spectra of photocatalysis of H_2O_2 from O_2 by a) and b) KMCN-G.

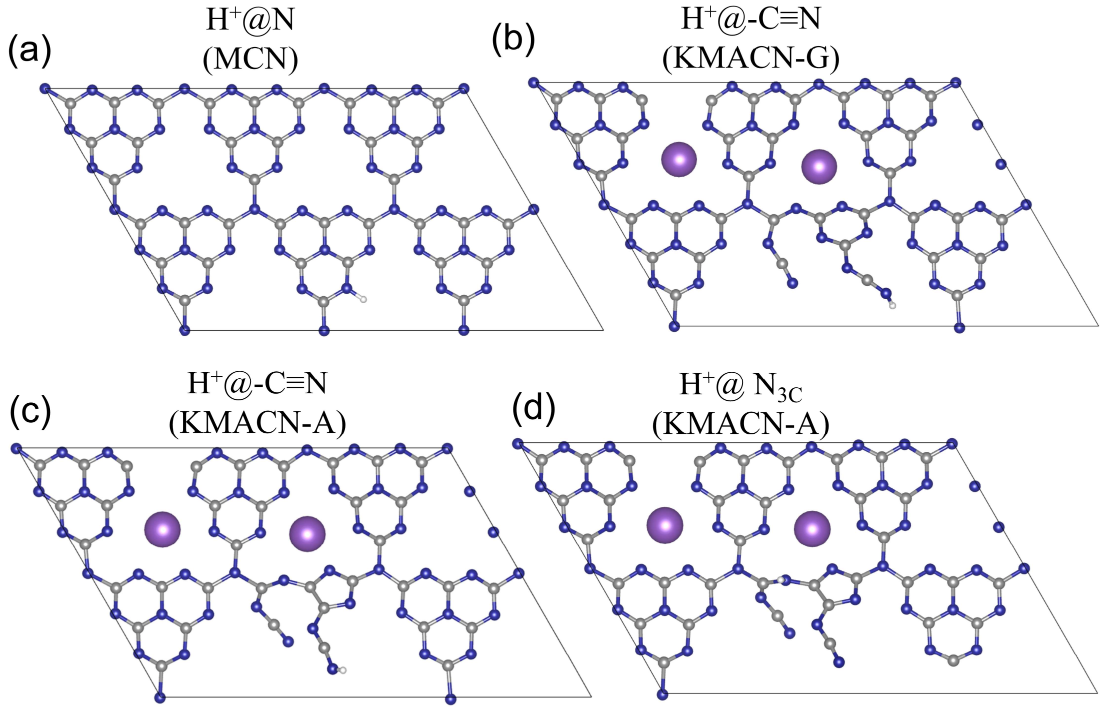

Figure S27. The position of H^+ adsorption and the corresponding H^+ adsorption energy on g-C_3N_4 models with different defect structures. The white spheres represent the adsorbed H^+.

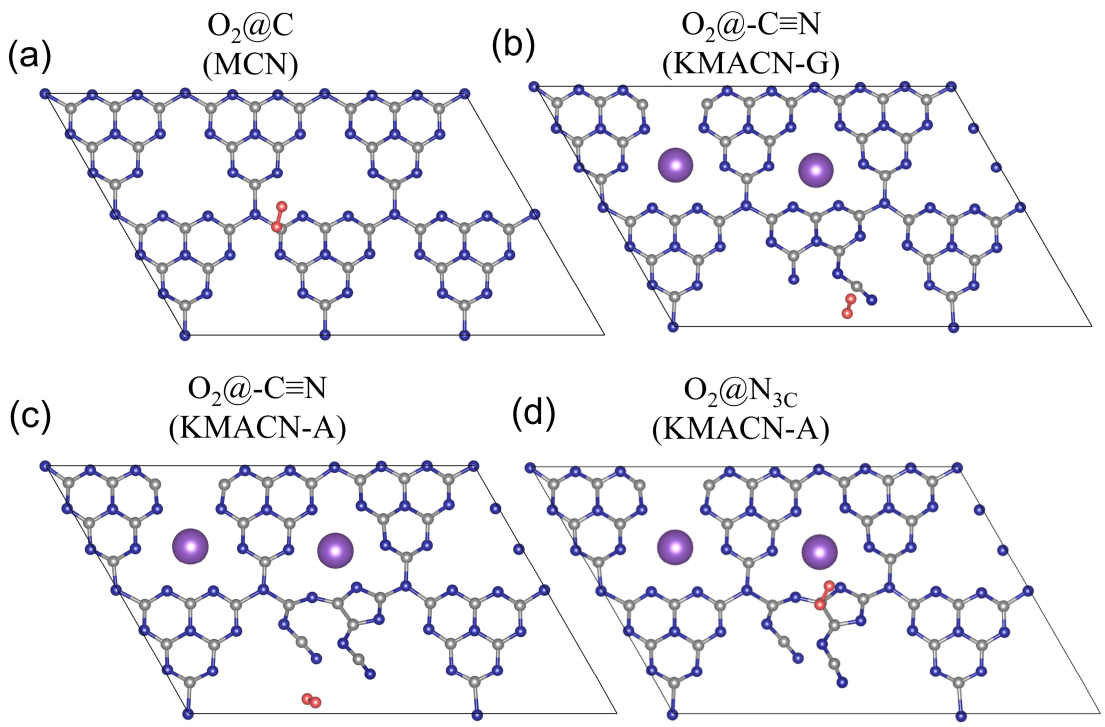

Figure S28. The position of O_2 adsorption and the corresponding O_2 adsorption energy on g-C_3N_4 models with different defect structures.

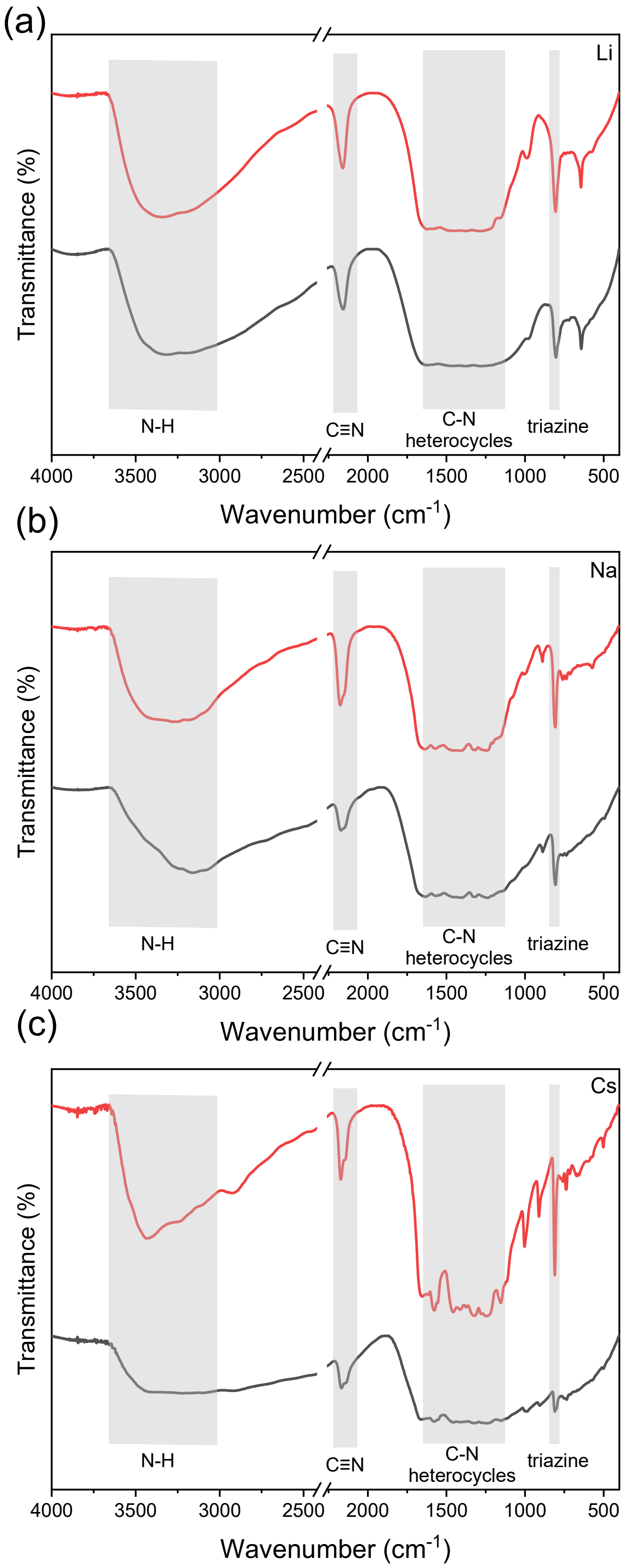

Figure S29. FT-IR spectrum of different elements CN catalysts (a)Li, (b)Na, and (c)Cs. prepared by different methods, physical grinding (black line); molecular assembly-molten salt coupling (red line)

Table S1. A literature survey for the production rate of H_2O_2 by different catalysts.

**Catalysts**	**Reactant Solution**	**Light Source**	**Rate of H_2O_2 production ($\mu mol \cdot g^{-1} \cdot h^{-1}$)**
P-mMCNNS[6]	5% ethanol	AM 1.5G	1083
DCN-15A[7]	20% Isopropanol	λ >420nm	96.8
g-C3N4-CNTs [8]	5% HCOOH	λ >400nm	326
Nv-0.05[9]	10% Isopropanol	λ ≥420 nm	623.5
KPD-CN-7.5[10]	5% ethanol	λ >320nm	466.7
Ni/OtCN[11]	10% ethanol	λ >420nm	2464
CoO_X-NvCN[12]	Pure water	λ >420nm	244.8
CKCN-0.03[13]	10% ethanol	AM 1.5G	610.4
KTTCN[14]	10% Isopropanol	λ >420nm	720
ANQ-POP[15]	10% ethanol	λ >400nm	800
TBCN@rGO[16]	5% ethanol	λ >420nm	172
Cu(2)-SCN[17]	Pure water	λ >400nm	266.7
CdS-PDA[18]	Pure water	λ >420nm	160
CDsMCN[19]	10% Isopropanol	λ >420nm	1480
DDCN[20]	5% methanol	λ >420nm	1031
KMACN-A (This work)	10% lactic acid	λ >420nm	2650.7

Table S2. FWHM values of (002) crystal planes in Figure 2a.

**Catalysts**	**FWHM (002)**
MCN	1.365
MACN	1.537
KMCN-G	1.367
KMACN-G	2.378
KMACN-A	1.970

Table S3. XPS surface atoms ratio and N/C.

**Catalysts**	**N/C**	**N_{2C}/C**	**N_{3C}/C**	**NH_X/C**	**N_{2C}/N_{3C}**
MCN	1.04	0.69	0.22	0.09	3.14
MACN	1.33	0.92	0.20	0.12	4.60
KMCN-G	1.18	0.83	0.16	0.11	5.18
KMACN-G	1.16	0.83	0.15	0.13	5.53
KMACN-A	0.96	0.66	0.12	0.12	5.50

Table S4. XPS surface total atom ratio

**Catalysts**	**C**	**N**	**O**	**K**
MCN	46.60	48.37	5.02	/
MACN	42.21	56.19	1.59	/
KMCN-G	43.52	51.62	1.81	3.05
KMACN-G	43.04	49.89	3.75	3.32
KMACN-A	45.8	44.02	5.19	5.02

Table S5. The fitted parameters in EIS.

**Catalysts**	R_{ct} (Ω)
MCN	270880
KMCN-G	131810
KMACN-G	91086
KMACN-A	48557

References

[1] J. Cai, J. Huang, A. Cao, Y. Wei, H. Wang, X. Li, Z. Jiang, G. I. N. Waterhouse, S. Lu, S.-Q. Zang, *Applied Catalysis B: Environmental* **2023**, *328*, 122473.

[2] Y. Lu, Y. Guo, S. Zhang, L. Li, R. Jiang, D. Zhang, J. C. Yu, J. Wang, *ACS Nano* **2024**.

[3] N. H. Anh, D.-V. Nguyen, T. A. Luu, P. D. M. Phan, H. P. Toan, P. P. Ly, N. Q. Hung, N. L. Nguyen, S. H. Hur, P. T. Hue, N. T. N. Hue, M.-T. Pham, T. D. T. Ung, D. D. Bich, V.-A. Dao, H. V. Doan, M. Isaacs, M. C. Nguyen, W. J. Yu, Y.-Y. Lee, G.-P. Chang-Chien, H.-T. Vuong, *Solar RRL* **2024**, *8*, 2400034.

[4] S. Samanta, R. Yadav, A. Kumar, A. Kumar Sinha, R. Srivastava, *Applied Catalysis B: Environmental* **2019**, *259*, 118054.

[5] S. Wu, H. Yu, S. Chen, X. Quan, *ACS Catal.* **2020**, *10*, 14380.

[6] L. Zhou, J. Feng, B. Qiu, Y. Zhou, J. Lei, M. Xing, L. Wang, Y. Zhou, Y. Liu, J. Zhang, *Applied Catalysis B: Environmental* **2020**, *267*, 118396.

[7] L. Shi, L. Yang, W. Zhou, Y. Liu, L. Yin, X. Hai, H. Song, J. Ye, *Small* **2018**, *14*, 1703142.

[8] S. Zhao, T. Guo, X. Li, T. Xu, B. Yang, X. Zhao, *Applied Catalysis B: Environmental* **2018**, *224*, 725.

[9] J. Li, J. Huang, G. Zeng, C. Zhang, H. Yu, Q. Wan, K. Yi, W. Zhang, H. Pang, S. Liu, S. Li, W. He, *Chem. Eng. J.* **2023**, *463*, 142512.

[10] G. Moon, M. Fujitsuka, S. Kim, T. Majima, X. Wang, W. Choi, *ACS Catal.* **2017**, *7*, 2886.

[11] R. Du, K. Xiao, B. Li, X. Han, C. Zhang, X. Wang, Y. Zuo, P. Guardia, J. Li, J. Chen, J. Arbiol, A. Cabot, *Chem. Eng. J.* **2022**, *441*, 135999.

[12] J. Hou, K. Wang, X. Zhang, Y. Wang, H. Su, C. Yang, X. Zhou, W. Liu, H. Hu, J. Wang, C. Li, P. Ma, R. Zhang, Z. Wei, Z. Sun, Q. Liu, K. Zheng, *ACS Catal.* **2024**, *14*, 10893.

[13] Y. Xie, Y. Li, Z. Huang, J. Zhang, X. Jia, X.-S. Wang, J. Ye, *Applied Catalysis B: Environmental* **2020**, *265*, 118581.

[14] J. Zhang, **2020**.

[15] B. Boro, N. Kim, J.-S. Kim, R. Paul, Y. Nailwal, Y. Choi, D.-H. Seo, J. Mondal, J. Ryu, *Journal of Colloid and Interface Science* **2023**, *652*, 1784.

[16] A. Behera, A. K. Kar, R. Srivastava, *Inorg. Chem.* **2022**, *61*, 12781.

[17] S. Hu, X. Qu, P. Li, F. Wang, Q. Li, L. Song, Y. Zhao, X. Kang, *Chemical Engineering Journal* **2018**, *334*, 410.

[18] Z. Wei, S. Zhao, W. Li, X. Zhao, C. Chen, D. L. Phillips, Y. Zhu, W. Choi, *ACS Catal.* **2022**, *12*, 11436.

[19] H. Guo, L. Zhou, K. Huang, Y. Li, W. Hou, H. Liao, C. Lian, S. Yang, D. Wu, Z. Lei, Z. Liu, L. Wang, *Advanced Functional Materials* **2024**, *n/a*, 2402650.

[20] G. Ba, H. Hu, F. Bi, J. Yu, E. Liu, J. Ye, D. Wang, *Applied Catalysis B: Environment and Energy* **2025**, *361*, 124645.
